# Supplementary material for: Campylobacter occurrence and antimicrobial resistance profile in under five-year-old diarrheal children, backyard farm animals, and companion pets
Source: PLoS Negl Trop Dis. 2024 Jun 4;18(6):e0012241. doi: 10.1371/journal.pntd.0012241 (PMC11178231; doi:10.1371/journal.pntd.0012241)
Supplement: Attachment — Submitted filename: Reviewer feedback T-PLOS-2024.pdf [file pntd.0012241.s006.pdf]

Reviewer t

*The authors conducted a study with a title on "***Campylobacter* **in children under the age of five with diarrhea, backyard farm animals, and companion animals: Occurrence and antibiotic resistance"** *in Bahirdar, Ethiopia.*

The study participants were basically under five children who had diarrhea (n=303) and had visited one of the several health centers in Bahirdar to receive medical attention. Furthermore, the survey encompassed farm animals and companion pets (n = 711) in the children's household's level.

The main results showed that the prevalence of campylobacteriosis was 20% in children who had diarrhea and roughly 22% in all farm and companion animals, with dogs and poultry having the highest rates (64%) and 44%, respectively. MICs determined using ETest showed 20% and 11% resistance to ciprofloxacin and doxycycline, respectively; whereas 0.8% of tested C. jejuni isolates were resistant to erythromycin and azithromycin.

The use of a large sample size and the membrane filtration culture technique for campylobacter isolation, together with the use of Etest strips and MALDI-TOF MS, were highlighted by the authors as the study's strong points. However, the microaerobic condition without a hydrogen-enriched atmosphere used limited the isolation of *Campylobacter* species other than *C. jejuni* and *C. coli*.

General comment

- In general, I have seen that the manuscript contains substantial research that is adequate for publishing.
- The paper may require language edition. The manuscript would benefit from re-visiting it to a recently accepted manuscript template.
- While the broad/main objective has been clearly articulated in the **introduction** part, there are no specific objectives, research questions, and a hypothesis partly in the 'Abstract' and introduction. These are needed to guide the analyses you made as well as to anchor the results and discussion.

Specific

Title: Campylobacter in children under the age of five with diarrhea, backyard farm animals, and companion animals: Occurrence and antibiotic resistance

Suggest: **Occurrence and antibiotic resistance profile of Campylobacter among under five-year diarrheic children, farm- and companion- animals**

or

Campylobacter occurrence and antibiotic resistance profile in under-five-year-old diarrheal children, farm animals, and companion animals

Abstract

Suggest: developing country for "growing countries"

Suggest: Antimicrobial resistance (AMR) is preferable as it is used on page 4, second paragraph than Antibiotic resistance (ABR). Revise throughout to adjust uniformly to AMR since both are used in your manuscript.

Introduction:

1st paragraph first line the dot ('.') be changed to the acceptable comma (',') form "….. 525,000 deaths ….". There are similar patterns throughout and check if they have to be changed.

Page 6: first paragraph second line: citations for the standard guidelines are not indicated.

Study area and design

According to the details provided under the subtitle "Study participants," the study is conducted at a healthcare facility, and the participants were people seeking medical attention at one of the many Bahirdar healthcare facilities. Because most symptomatic instances of campylobacter may not seek medical attention, the study cannot be considered community-based.

Isolation and preliminary identification of *Campylobacter*

Page 7: The isolation of campylobacter was carried out in this subtitle for a maximum of five days at 37 degrees Celsius in a microaerobic atmosphere (burning candle). Under the same conditions, additional confirmation was carried out in an Ethiopian laboratory using the candle extinction method. Although the researchers used sophisticated identification techniques in Belgium and appropriate re-cultivation techniques for campylobacter, the initial isolation methods used in Ethiopia—that is, the candle extinction method for primary isolation versus the maintenance of microaerobic conditions through the introduction of a gas generator to provide a mixture of gases (10% CO2, 10% H2, and 80% N2) under the heading **"Re-cultivation of presumptive Campylobacter isolates"—**cannot provide confidence for the true rate of campylobacter positivity.

The initial measures taken to recover Campylobacter from the specimens of symptomatic children and the different animals revealed one of the study's major weaknesses. It is evident that the bacteria are very sensitive to even a small change in environmental factors. According to the majority of the literatures, microaerophilic bacteria, such as Campylobacters, can be grown in plastic bags or sealed jars using commercial gas-generating envelopes that contain mixes of CO_2, H_2, O_2, and N2.

Table 2: The 'Age' column addition is 57, and further the total children involved become 301. Where are the two positive results missed in the table2?

Table 4: Break point; 0.004

References (page 29-38): The number of references is too much. There is redundant citation of the same article in four places as can be checked from the PDF file.

1. Asuming-Bediako N, Parry-Hanson Kunadu A, Abraham S, Habib I. *Campylobacter* at the human–food interface: the african perspective. Pathog. 2019;8(2):87.

2. Asuming-Bediako N, Parry-Hanson Kunadu A, Abraham S, Habib I. *Campylobacter* at the human–food interface: the african perspective. Pathog. 2019;8(2):87.
3. Debelo M, Mohammed N, Tiruneh A, Tolosa T. Isolation, identification and antibiotic resistance profile of thermophilic *Campylobacter* species from Bovine, Knives and personnel at Jimma Town Abattoir, Ethiopia. PLoS One. 2022;17(10):e0276625.
4. Debelo M, Mohammed N, Tiruneh A, Tolosa T. Isolation, identification and antibiotic resistance profile of thermophilic *Campylobacter* species from Bovine, Knives and personnel at Jimma Town Abattoir, Ethiopia. PLoS One. 2022;17(10):e0276625.
5. Terefe Y, Deblais L, Ghanem M, Helmy YA, Mummed B, Chen D, et al. Co-occurrence of *Campylobacter* species in children from Eastern Ethiopia, and their association with environmental enteric dysfunction, diarrhea, and host microbiome. Front Public Health. 2020;8:99.
6. Terefe Y, Deblais L, Ghanem M, Helmy YA, Mummed B, Chen D, et al. Co-occurrence of *Campylobacter* species in children from Eastern Ethiopia, and their association with environmental enteric dysfunction, diarrhea, and host microbiome. Front Public Health. 2020;8:99.
7. Vasco K, Graham JP, Trueba G. Detection of zoonotic enteropathogens in children and domestic animals in a semirural community in Ecuador. Appl Environ Microbiol. 2016;82(14):4218-24.
8. Vasco K, Graham JP, Trueba G. Detection of zoonotic enteropathogens in children and domestic animals in a semirural community in Ecuador. Appl Environ Microbiol. 2016;82(14):4218-24.

One article may need to be checked for a full citation of the article: [Houf K. *Campylobacter* and *Arcobacter*. 2020.]

Referencing Manager engines may give reliable solutions to the above problems: including but not limited to avoid repetition and correctly mention the citations based on the publisher requirements.

Questions for consideration:

1. Incubation of cultured media for Campylobacter isolation use to be for 48 hours with additional 24 hours before it is recorded negative. However, in your study, it was extended for upt o five days. How much of your isolates were recovered by extending to 72 to 120 hours before it was defined as ‘No growth for Campy”? That part of knowledge is essential.
2. In discussion part, page 23, second paragraph last line: “…. the use of microaerobic conditions without hydrogen during isolation” was stated as a reason for failing to recover slow-growing campylobacter species. Don’t you believe that you also missed the human important fast growing campylobacter pathogens from your study? Which Campy species that may be relevant in your study may be missed by the absence of the gas as well?
3. Which statistical tools are used for which variables, parameters or factors cannot be appreciated in the manuscript. As some of the observed values are too small to extract meaningful interpretation and conclusion, a reliable professional consultation may be necessary.
