# Supplementary material for: Campylobacter occurrence and antimicrobial resistance profile in under five-year-old diarrheal children, backyard farm animals, and companion pets
Source: PLoS Negl Trop Dis. 2024 Jun 4;18(6):e0012241. doi: 10.1371/journal.pntd.0012241 (PMC11178231; doi:10.1371/journal.pntd.0012241)
Supplement: Attachment — Submitted filename: PNTD-D-23-01585 -Reviewed 2024.pdf [file pntd.0012241.s007.pdf]

# PLOS Neglected Tropical Diseases

## Campylobacter in children under the age of five with diarrhea, backyard farm animals, and companion animals: Occurrence and antibiotic resistance

--Manuscript Draft--

| | |
|---|---|
| **Manuscript Number:** | PNTD-D-23-01585 |
| **Full Title:** | Campylobacter in children under the age of five with diarrhea, backyard farm animals, and companion animals: Occurrence and antibiotic resistance |
| **Short Title:** | Occurrence and antibiotic resistance profiles of Campylobacter |
| **Article Type:** | Research Article |
| **Keywords:** | Campylobacter; children-animal interface; Membrane-filtration culture; MALDI-TOF |
| **Abstract:** | Background<br><br>Campylobacteriosis disproportionately affects children under five in growing countries. However, reliable epidemiological and antibiotic resistance (ABR) information at the children-animal interface is lacking. This study examined Campylobacter occurrence and its ABR in a large cohort of young children with acute diarrhea, backyard farm animals, and companion animals using new methodologies.<br><br>Methodology/Principal Findings<br><br>Via local health centers, stool from 303 children, and feces from 711 animals were sampled. Campylobacter were isolated through membrane filtration on modified charcoal cefoperazone deoxycholate agar plates under microaerobic incubation. Typical isolates were characterized with MALDI-TOF MS and multiplex PCR. Chi-square, Fisher's exact, Mann-Whitney U, Kruskal-Wallis, and post hoc statistical tests were applied to assess differences in occurrence. Of 303 children (median age 19 months (IQR, 12-31.5)), 20% (n = 59) were infected, with a higher proportion in the 6 to 11-month age group. The occurrence of Campylobacter was 64% (n = 14) in dogs and 44% (n = 112) in poultry. Campylobacter jejuni was identified in 19% (n = 194) of samples. Campylobacter jejuni was present in both a child and animal species in 15% (n = 23) of 149 households positive for Campylobacter. MICs from the gradient strip diffusion test of 128 isolates displayed resistance rates of 20% to ciprofloxacin and 11% to doxycycline. MICs of ciprofloxacin and doxycycline varied between C. coli and C. jejuni, with higher resistance in C. coli. Higher resistance was present in isolates from poultry.<br><br>Conclusions<br><br>Campylobacter infection in children and its prevalent excretion from backyard poultry and dogs is a understudied concern. The co-occurrence of C. jejuni in animals and children is suggestive of direct transmission. As resistance to ciprofloxacin and doxycycline was observed, therapy of severe campylobacteriosis should be guided by susceptibility testing. Filtration-culture is a feasible diagnostic technique for use in regions lacking organized laboratories. Findings from this study will support evidence-based diagnosis, antimicrobial treatment, and further investigations on the spread of antimicrobial resistance mechanisms for informed One Health intervention. |
| **Additional Information:** | |
| **Question** | **Response** |
| **Government Employee**<br><br>Are you or any of the contributing authors an employee of the United States government? | No - No authors are employees of the U.S. government. |

| | |
|---|---|
| Manuscripts authored by one or more US Government employees are not copyrighted, but are licensed under a CC0 Public Domain Dedication, which allows unlimited distribution and reuse of the article for any lawful purpose. This is a legal requirement for US Government employees.<br><br>This will be typeset if the manuscript is accepted for publication. | |
| **Financial Disclosure**<br><br>Enter a financial disclosure statement that describes the sources of funding for the work included in this submission. Review the submission guidelines for detailed requirements. View published research articles from *PLOS Neglected Tropical Diseases* for specific examples.<br><br>This statement is required for submission and **will appear in the published article** if the submission is accepted. Please make sure it is accurate.<br><br>**Funded studies**<br>Enter a statement with the following details:<br>• Initials of the authors who received each award<br>• Grant numbers awarded to each author<br>• The full name of each funder<br>• URL of each funder website<br>• Did the sponsors or funders play any role in the study design, data collection and analysis, decision to publish, or preparation of the manuscript?<br><br>Did you receive funding for this work? | Yes |
| Please add funding details.<br>as follow-up to "**Financial Disclosure**<br><br>Enter a financial disclosure statement that describes the sources of funding for the work included in this submission. Review the submission guidelines for detailed | 1/ Research fund of the Department of Medical Laboratory Science, College of Medicine and Health Sciences, Bahir Dar University, Bahir Dar, Ethiopia<br><br>2/ Research grant for Ghent university, Belgium |

| | |
|---|---|
| requirements. View published research articles from *PLOS Neglected Tropical Diseases* for specific examples.<br><br>This statement is required for submission and **will appear in the published article** if the submission is accepted. Please make sure it is accurate.<br><br>**Funded studies**<br>Enter a statement with the following details:<br>• Initials of the authors who received each award<br>• Grant numbers awarded to each author<br>• The full name of each funder<br>• URL of each funder website<br>• Did the sponsors or funders play any role in the study design, data collection and analysis, decision to publish, or preparation of the manuscript?<br><br>Did you receive funding for this work?" | |
| Please select the country of your main research funder (please select carefully as in some cases this is used in fee calculation).<br>as follow-up to "**Financial Disclosure**<br><br>Enter a financial disclosure statement that describes the sources of funding for the work included in this submission. Review the submission guidelines for detailed requirements. View published research articles from *PLOS Neglected Tropical Diseases* for specific examples.<br><br>This statement is required for submission and **will appear in the published article** if the submission is accepted. Please make sure it is accurate. | ETHIOPIA - ET |

| | |
|---|---|
| **Funded studies**<br>Enter a statement with the following details:<br>• Initials of the authors who received each award<br>• Grant numbers awarded to each author<br>• The full name of each funder<br>• URL of each funder website<br>• Did the sponsors or funders play any role in the study design, data collection and analysis, decision to publish, or preparation of the manuscript?<br><br>Did you receive funding for this work?" | |
| **Competing Interests**<br><br>On behalf of all authors, disclose any competing interests that could be perceived to bias this work.<br><br>This statement will be typeset if the manuscript is accepted for publication.<br><br>Review the instructions link below and PLOS NTDs' competing interests policy to determine what information must be disclosed at submission. | none |
| **Data Availability**<br><br>Provide a **Data Availability Statement** in the box below. This statement should detail where the data used in this submission can be accessed. This statement will be typeset if the manuscript is accepted for publication.<br><br>Before publication, authors are required to make all data underlying their findings fully available, without restriction. Review our PLOS Data Policy page for detailed information on this policy. Instructions for writing your Data Availability statement can be accessed via the Instructions link below. | All raw and analysed data are available in the e-book of the first author. Isolates have been depositit in the LMG-UGent reserach collection and are availble on request |

Powered by Editorial Manager® and ProduXion Manager® from Aries Systems Corporation

Manuscript Click here to access/download;Manuscript;Manuscript.pdf

# *Campylobacter* in children under the age of five with diarrhea, backyard farm animals, and companion animals: Occurrence and antibiotic resistance

Wondemagegn Mulu[1,2], Marie Joossens[1], Mulugeta Kibret[3], Anne-Marie Van den Abeele[4,5], Kurt Houf [1,5*]

[1]Laboratory of Microbiology, Department of Biochemistry and Microbiology, Ghent University, Ghent, Belgium

[2] Department of Medical Laboratory Science, College of Medicine and Health Sciences, Bahir Dar University, Bahir Dar, Ethiopia

[3] Department of Biology, Science College, Bahir Dar University, Bahir Dar, Ethiopia

[4] Laboratory of Microbiology, Sint-Lucas Hospital, Ghent, Belgium

[5] Department of Veterinary and Biosciences, Faculty of Veterinary Medicine, Ghent University, Ghent, Belgium

[*]Corresponding author

Email: kurt.houf@ugent.be

**Short title: Occurrence and antibiotic resistance profiles of *Campylobacter***

**Abstract**

**Background**: Campylobacteriosis disproportionately affects children under five in growing countries. However, reliable epidemiological and antibiotic resistance (ABR) information at the children-animal interface is lacking. This study examined *Campylobacter* occurrence and its ABR in a large cohort of young children with acute diarrhea, backyard farm animals, and companion animals using new methodologies.

**Methodology/Principal Findings**: Via local health centers, stool from 303 children, and feces from 711 animals were sampled. *Campylobacter* were isolated through membrane filtration on modified charcoal cefoperazone deoxycholate agar plates under microaerobic incubation. Typical isolates were characterized with MALDI-TOF MS and multiplex PCR. Chi-square, Fisher's exact, Mann-Whitney U, Kruskal-Wallis, and post hoc statistical tests were applied to assess differences in occurrence. Of 303 children (median age 19 months (IQR, 12-31.5)), 20% (n = 59) were infected, with a higher proportion in the 6 to 11-month age group. The occurrence of *Campylobacter* was 64% (n = 14) in dogs and 44% (n = 112) in poultry. *Campylobacter jejuni* was identified in 19% (n = 194) of samples. *Campylobacter jejuni* was present in both a child and animal species in 15% (n = 23) of 149 households positive for *Campylobacter*. MICs from the gradient strip diffusion test of 128 isolates displayed resistance rates of 20% to ciprofloxacin and 11% to doxycycline. MICs of ciprofloxacin and doxycycline varied between *C. coli* and *C. jejuni*, with higher resistance in *C. coli*. Higher resistance was present in isolates from poultry.

**Conclusions**: C*ampylobacter* infection in children and its prevalent excretion from backyard poultry and dogs is a understudied concern. The co-occurrence of *C. jejuni* in animals and children is suggestive of direct transmission. As resistance to ciprofloxacin and doxycycline was observed, therapy of severe campylobacteriosis should be guided by susceptibility testing. Filtration-culture is a feasible diagnostic technique for use in regions lacking organized laboratories. Findings from this study will support evidence-based diagnosis, antimicrobial treatment, and further investigations on the spread of antimicrobial resistance mechanisms for informed One Health intervention.

**Keywords**: *Campylobacter*, children-animal interface, Membrane-filtration culture, MALDI-TOF MS, Multiplex PCR, MIC, Gradient strip diffusion, Ethiopia

## Author Summary

Diarrheal diseases are the second-leading cause of death in children under five, and *Campylobacter* is a major global causative agent. However, little attention is given to the accurate isolation, characterization, and antibiotic resistance assessment of *Campylobacter* in sub-Saharan Africa. This region has the highest burden of diarrhea but a lack of organized laboratories. This study investigated the presence and antibiotic resistance of *Campylobacter* in children and animals using methods with minimal technical challenge. We collected stool samples from 303 children at health centers and 711 animal feces samples from the children's homes in the vicinity of Bahir Dar, Ethiopia. *Campylobacter* were isolated using membrane filtration-culture. Presumptive isolates were characterized with molecular techniques in Belgium. Membrane filtration culture with a traditional microaerobic incubation system was successful in isolating viable *Campylobacter* from children with diarrhea and animals. *Campylobacter* is highly prevalent in children, backyard poultry, and dogs and has shown resistance to ciprofloxacin and doxycycline. The key findings of this study clearly show that *Campylobacter* infection in children and its occurrence in animals associated with children is a major public health problem. These findings are supportive of clinical decision-making and treatment. Findings from this study are also relevant as a validation of applying the membrane filtration-isolation technique for *Campylobacter* screening in growing countries.

## Introduction

Gastrointestinal diseases cause around 525.000 deaths yearly in children under five [1] and *Campylobacter jejuni* and *Campylobacter coli* have been ranked as one of the four principal microorganisms causing gastroenteritis worldwide [2, 3]. Campylobacteriosis occurs in all age groups in high-income countries, but it is widespread and disproportionately affects children under five in low-income countries [4]. A pooled prevalence of 10% was reported in children under five, and 9% in a general population in Ethiopia [5, 6]. In children under five, it is strongly linked to severe morbidity, environmental enteric dysfunction, malnutrition, failure to thrive, neurological disorders and death [7, 8]. Children afflicted with campylobacteriosis experience fever, vomiting, abdominal cramps, watery to severe inflammatory diarrhea and dehydration [9]. Furthermore, post-infection complications of campylobacteriosis include arthritis, Guillain-Barré syndrome resulting in respiratory and neurological dysfunction, and irritable bowel syndrome [10, 11].

Ciprofloxacin is one of the first choice treatment options in severe cases of bacterial enteritis [12]. Currently, children are however mostly treated without prior identification of the specific causative organism and antibiotic susceptibility profiling in Ethiopia [13]. The use of inappropriate antibiotics for empirical treatment might contribute to the selection of resistant strains and antimicrobial resistance. Antimicrobial resistance (AMR) can result from a genetic mutation within the organism or acquiring resistance genes from other organisms following selection pressures in humans and animals [14].

*Campylobacter* can colonize, persist, and exert different pathogenicity in a variety of hosts, and is considered the main cause of foodborne bacterial zoonotic infections [15]. Poultry, pigs, and cattle are important reservoirs for the thermophilic *Campylobacter* species, *C. jejuni* and *C. coli* [16, 17]. Direct contact with feces of poultry, ruminants, and pets, as well as consumption of contaminated food of animal origin and drinking water, are considered the main transmission routes [18, 19]. Due to the broad host range and uncontrolled uses of antibiotics in human and veterinary medicine, and agriculture, the emergence and dissemination of AMR in *Campylobacter* is both a global health challenge and threat to One Health [20-23].

*Campylobacter* is a Gram-negative curved or spiral, motile, microaerobic and fastidious bacterium, and not easy to cultivate in sub-optimal laboratory conditions, as in some parts of Ethiopia [2, 3]. As a result, insufficient attention is given to the epidemiological burden of disease

and to optimal isolation and correct identification of *Campylobacter* from children with diarrhea. Because of variability in isolation procedures with respect to the choice of culture media and systems of microaerobic condition, temperature, and duration of incubation, results from previous studies are inconsistent. Aside from two recent studies, Terefe et al. [24] and Chala et al. [23], which applied molecular methods to detect *Campylobacter* in stool, the majority of Ethiopian studies have been solely performed by classical and biochemical identification methods. Both studies using molecular tools, included limited numbers of samples (less than 100), and each was carried out in a single study center [23, 24]. Also, studies involving humans, poultry, and/or cattle from the same households are rare, participate small sample sizes (total number of human and animal samples combined: n = 347 and 70) [23, 25], and describe individuals of not age specific [23] and infants [25], regardless of the status of their diarrhea.

*Campylobacter* exposure to antibiotics is expected to be high. Inappropriate empirical treatment of enteritis in children is common in the study area, and resistant infections result in toxicity, increased morbidity and mortality rates, and a heightened cost of care [14]. Up till now, data on *Campylobacter* enteritis and antibiotic susceptibility testing (AST) profiles in children with acute diarrhea in homes with backyard farm animals and/or companion animals is expected to be inaccurate in Ethiopia, due to the use of difficult to compare and/or suboptimal methods for isolation and AST.

Around 85% of rural households in Ethiopia own cattle, sheep, goats, poultry, and pets which interact closely with family members in the residences [26]. They are frequently kept indoors and roam freely so that animal feces are present in-house. As a result, young children can acquire *Campylobacter* directly from animals or surfaces contaminated with animal waste. However, knowledge on the occurrence of *Campylobacter* between children under five and animals is lacking in Ethiopia. Due to the lack of toilet facilities, human defecation in the open, close to homes and drinking water sources, is also a common practice. Therefore, assessing the presence of *Campylobacter* in children and animals, along with their susceptibility towards locally available antibiotics is highly relevant.

Therefore, the present study aimed to assess the occurrence of *Campylobacter* in a large cohort of children under the age of five years with diarrhea, as well as backyard farm animals and companion animals associated with these children. It also aimed to determine the antibiotic

resistance profiles of *Campylobacter* isolates from children and animals to antibiotics locally prescribed or available and recommended by standard guidelines (?). New methodologies were used in order to generate baseline data that could support clinical-decision making and treatment and provide insights on *Campylobacter* transmission and antibiotic resistance between children and animals for the overall mitigation of campylobacteriosis and AMR.

## Materials and Methods

### Study participants

Children under five suffering from diarrhea, who sought treatment at the health centers involved, and from whom sufficient feces for sample collection could be collected, were eligible to participate. Children were eligible to participate upon written parental consent. Children experiencing diarrhea for more than seven days and coming from a household without any backyard farm animals were excluded.

How about pet animals while farm animals are absent?

### Study area and design

From the above information, it is healthcare facility based research where the study participants were health seekers coming to the health centers, yes?

A community-based cross-sectional study was conducted in the surroundings of Bahir Dar City, Ethiopia, from March 15, 2021, to April 15, 2022. Bahir Dar City is located at the point where the Blue Nile River leaves Lake Tana and at an altitude of 1820 m (GPS coordinates of 11° 34' 27.1524" N and 37° 21' 40.8708" ?E or W) (Fig 1A) [27]. Children with diarrhea were recruited from Kinbaba, Robit, Wonjeta and Yinesa Health centers in Bahir Dar Zuria, and from Abay, Han and Zege Health Centers in Bahir Dar City (Fig 1B). Health Centers that were logistically difficult to access and had only few children with diarrhea during the collection period of the current study were excluded. Based on the data received from the administrative offices, Kinbaba, Wonjeta, Yinesa and Robit Health Centers provided health care services for 32.668, 42.898, 17.303 and 28.401 people, respectively. Han, Zege and Abay Health Centers served 9.354, 5.025 and 10.306 people, respectively. The houses visited in this Bahir Dar City recruitment area were in a radius of 0.4 to 12.8 kilometers from the health centers. In Kinbaba, the houses visited were in a 1.5 to 12.8 km radius from the health center; in Yinesa, they were in a 1.7 to 9.2 km radius; and in Wonjeta, they were in a 1.3 to 8.3 km radius.

Relevance of mentioning this doesn't show benefit!

You may replace the blue highlights with "From each of the health centers within a 0.4 to 12.8 KM radius, every household with the suspected cases was visited."

**Stool sample and data collection from children**

Samples were collected at each health center and 5- 10 grams stool per child were transferred in a sterile plastic cup for storage [25], labeled with child code and date of collection. Samples were kept refrigerated at the health centers until transportation to the microbiology laboratory at Bahir Dar University within 4 hours of collection. Along with the sample, data on children's age, sex, residence, and clinical details (fever, abdominal pain, vomiting, duration of diarrhea, and frequency of passing stool per 24 h) were recorded from each child during face-to-face interviews with their parent(s) according to the approved questionnaire.

Where is the tool? Which important variables are otherwise addressed in the tool?

**Fecal sample collection from animals**

Following the sampling of the children at the health centers, a visit to their respective homes was organized within one week. From the animals present at home, about 25 grams of fresh feces from bovines, sheep, and goats were collected using a clean spoon-attached stool cup [23]. In case of lack of spontaneous defecation from the livestock, feces were collected from the rectum [28]. For poultry, 10-15 grams of fresh cecal droppings were pooled from the poultry houses. Finally, the family's indoor garden was observed for freshly excreted feces from pets.

Was there a kind of pooling fecal samples for the respective animal species type per household? Or did you use a different sampling approach to select the animals in a household?

**Isolation and preliminary identification of *Campylobacter***

Standard protocols (which ??) were followed for transportation, isolation, and preservation of isolates. From each stool and fecal sample, (about) 1 gram was diluted (homogenized) in 10 ml of sterile physiological water and gently vortexed for 30 seconds. A polycarbonate filter of 0.6-μm pore size (Global Life Sciences Solutions Operations (Ltd.) Little Chalfont Buckinghamshire, UK) was placed onto a modified charcoal cefoperazone deoxycholate agar plate (*Campylobacter* Blood-Free Selective Agar Base, CM0739, Oxoid, Basingstoke, UK) [29]. Six drops of the fecal suspension were added on top of a filter membrane and allowed to pass passively for 30 minutes at 37°C, ambient air [30, 31]. After filtration, the filter was removed, and plates were incubated in a glass jar for 48 to 120 h at 37°C under microaerobic atmosphere (burning candle) [18, 32]. Plates were checked after 48, 72, 96, and 120 h of incubation for the growth of typical *Campylobacter* colonies.

Preliminary identification of *Campylobacter* isolates was made by typical colony appearances on mCCDA plates. Translucent, grey-white with a metallic sheen, watery and spread- like droplets to a continuous film (swarming) colony, were considered presumptive *Campylobacter* colonies [33].

Subsequently, after microscopic confirmation of the presence of fine and spiral rods by safranin staining [16, 29], typical colonies were streaked onto a new mCCDA plate, and incubated for 24-72 h at 37 °C, under microaerobic conditions. Performance of mCCDA, at 37°C, and microaerobic incubation conditions were tested using *Campylobacter* reference strains from the Belgian Coordinated Collections of Microorganisms (BCCM/LMG).

**Storage and transportation of presumptive *Campylobacter* isolates**

Upon growth, pure colonies with typical *Campylobacter* morphology were swabbed from the second mCCDA plates with a sterile cotton swab, transferred to cryotubes (Thermo Scientific, Suxhou, Jiangsu, China) with 1 ml sterile sheep blood and stored at - 80°C. An additional aliquot was kept at -20 °C in Eppendorf tubes with 1 ml of sterile physiological water. The presumptive *Campylobacter* isolates were transported on dry ice from Ethiopia to the Microbiology Laboratory (LMG) at Ghent University, Belgium.

**Re-cultivation of presumptive *Campylobacter* isolates**

In Belgium, the suspensions were sub-cultured on blood agar (BA) plates (Columbia Agar Base, CM0331, Oxoid, with 5% defibrinated horse blood (E & O Laboratories Limited, Burnhouse, Scotland)) [32, 34]. In addition, 0.5 ml of each cell suspension was transferred to 10 ml of Preston Broth (PB) (Nutrient Broth No. 2, CM0067, Oxoid, with 2ml of sterile distilled water and Modified Preston *Campylobacter* Selective Supplement, SR0204E, Oxoid, and 5% lysed horse blood) in a sterile screw-capped bottle [35]. Blood agar plates and PB tubes were incubated at 37°C in microaerobic conditions by introducing a gas mixture (10% $CO_2$, 10% $H_2$ and 80% $N_2$) into metal jars. After 24 h of incubation, 10 µl of each enrichment broth was spread onto a BA plate and incubated for 48 h as described above [29]. The remaining PB tubes were incubated again for 24 h. Of samples for which no growth was obtained after 24 h, another 10 µl was spread from the corresponding PB culture onto a BA plate and incubated for 48-120 hrs [35]. Typical *Campylobacter* growth on the BA plates was harvested for further confirmation and identification at the species level using MALDI-TOF MS.

***Campylobacter* species identification by MALDI-TOF MS**

Typical *Campylobacter* colonies from BA plates were analyzed, mass spectra (MSP) generated and identified by MALDI-TOF MS (Bruker's MALDI-TOF Microflex LT/SH LMGE-0422) as

described previously [36, 37]. Interpretations of results were made by comparing the generated MSP Bruker log scores to the Bruker database MSP-10833 and LM-UGent iD MSP-4778 database using the Bruker Compass software (Bruker Daltonics). To ensure that the run was valid, calibration was performed before each set of measurements using 1µl Bacterial Test Standard (BTS) (Bruker Daltonics). Identification of *Campylobacter* at the species level was considered if scores were $\geq$ 2.2, according to the Bruker MALDI Biotyper specifications. Isolates that could not be re-cultivated, or yielded a score $<$ 2.2 identified, were further analyzed by PCR.

**PCR-based analysis of genus and species of *Campylobacter* -------------- *alternative subtitle***

### *Campylobacter* genus and species PCR-based analysis

DNA extraction from isolates was performed using the alkaline lysis method [38], and a *Campylobacter* genus-specific PCR was applied, targeting the 23S rRNA gene using the primer set THERM1 (5'-TATTCCAATACCAACATTAGT-3') and THERM2, (5'-CGGTACGGGCAACATTAG-3') (Merck Sigma-Aldrich, Belgium) [39]. The PCR reaction was carried out in a final reaction of 25 µl. Per reaction, 2 µl of the DNA template was used along with PCR buffer (Tris-Cl + KCl + $(NH4)_2$ SO4 + 15mM MgCl2) (QIAGEN), a deoxynucleotide triphosphate (dNTP) mixture (200 µM each), THERM1 primer (0.5 µM), THERM2 primer (0.5 µM), $MgCl_2$ (2 mM) and *Taq* polymerase (0.5 U/µl).

For all amplification experiments, the Veriti™ 96-Well Fast Thermal Cycler (Applied Biosystems™) was used with 27 amplification cycles, as described previously [39]. PCR products were size-separated by 1% (w/v) agarose gel electrophoresis in 1% Tris-borate-EDTA (TBE) buffer (VWR LIFE SCIENCE). Thermophilic *Campylobacter* strains gave amplicons corresponding to 290 bp [39]. The negative control yielded no amplification product.

Samples for which a *Campylobacter* genus-specific amplicon of 290 bp was generated, were subsequently analyzed in a *C. jejuni*/*C. coli* species-specific PCR assay. The primer sets mDmapA1, (5'-CTATTTTATTTTTGAGTGCTTGTG-3') and mDmapA2, (5'-GCTTTATTTGCCATTTGTTTTATTA-3') were applied targeting the map A gene from *C. jejuni*, and COL3, 5'-AATTGAAAATTGCTCCAACTATG-3' and MDCOL2, 5'-TGATTTTATTATTTGTAGCAGCG-3' (Merck Sigma-Aldrich) targeting the ceuE gene from *C. coli* (41). The PCR reaction was carried out in a final reaction of 25 µl. Per reaction, 2 µl of the DNA template was used along with PCR buffer (Tris-Cl + KCl + $(NH4)_2$ SO4 + 15mM $MgCl_2$) (QIAGEN), a dNTPs (200 µM each), $MgCl_2$ (2 mM), MDmapA1 (0.5 µM), MDmapA2 (0.5 µM),

COL3 (0.5 µM) and MDCol2 0.5 µM) and *Taq* (0.5 U/µl). Amplification reactions were performed using the Veriti™ 96-Well Fast Thermal Cycler (Applied Biosystems™) according to the program cycle described previously [40]. Amplification generated 589 bp and 462 bp DNA fragments for *C. jejuni* and *C. coli*, respectively.

**Antibiotic susceptibility testing**

The antibiotic susceptibility of *C. jejuni* and *C. coli* to ciprofloxacin, doxycycline, erythromycin, and azithromycin was determined using the Epsilometer gradient strip diffusion method (ETEST) (bioMérieux, Marcy-l'Etoile, France) on Mueller-Hinton Fastidious (MHF) agar (bioMérieux, Marcy-l'Etoile, France). Inocula were prepared by suspending colonies in MHF broth to a 0.5 McFarland density using a nephelometer. Inoculated plates were incubated under micro-aerophilic conditions using a gas mixture of 6% $O_2$, 7.1% $CO_2$, 7.1% $H_2$, and 79.7% $N_2$ (Anoxomat ™) at 37°C. After 48 h of incubation, the MIC values were read and interpreted based on the 2023 EUCAST Clinical Breakpoint Tables V.13.0 [41]. *Campylobacter jejuni* ATCC 33560 quality control strain was included for each separate run. Antibiotics were selected based on availability and prescription in the study area, and standard treatment and AST guidelines. Ciprofloxacin is the first choice of treatment for enteritis in the study area. In addition, ciprofloxacin and azithromycin are listed as first and second choices for enteritis treatment in primary care. All four antibiotics tested are locally available.

**Statistical analysis**

GPS coordinates of the visited homes were recorded, and their spatial distributions were visualized using ArcGIS version 10.5 software. Data were analyzed using IBM SPSS Statistics for Windows, Version 29.0. Armonk, NY: IBM Corp. SYSTAT. Descriptive statistics were used to summarize the data. Both the Shapiro-Wilk and Kolmogorov-Smirnov tests were used to test the normality of continuous data. When data were not normally distributed, the median and interquartile range (IQR) Q1 to Q3 were reported. For these data, Mann-Whitney U Test and Kruskal-Wallis Test were used to assess differences. Pearson Chi-Square tests were used to observe the association between *Campylobacter* infection and categorical variables. Fisher's exact test was used for categorical variables that had less than five observed values. Additionally, Chi-Square test was computed to compare the rate of antibiotic resistance. Games-Howell post-

hoc test was used to identify among which specific age groups the significant difference in *Campylobacter* infection occurs. P-values less than 0.05 were considered statistically significant.

**Ethical Statement**

The Institutional Review Board of the College of Medicine and Health Sciences (CMHS) of Bahir Dar University reviewed and approved the study protocols with reference number CMHS/IRB 01-008. A formal letter of support was obtained from Bahir Dar University CMHS and Amhara Public Health Institute. Data and samples were collected after receiving written informed consent. Health centers were reached through Bahir Dar City and Bahir Dar Zuria Health Departments. All survey, observation, and laboratory analysis data were anonymized, numbered, coded, and kept strictly confidential. For the proper management of patients, *Campylobacter* positive results were conveyed to nurses working at the health centers. Members of the households were informed about good practices in sanitation and hygiene to prevent *Campylobacter* infection and diarrhea. After authorization from the Ethiopian Food and Drug Authority, colonies presumptive of *Campylobacter* were transported from Bahir Dar, Ethiopia to Ghent, Belgium following national and international specimen transfer rules and regulations.

## Results

### Occurrence and identification of *Campylobacter*

As shown in Table 1, 303 children from 303 different households participated in the study and provided a fecal sample. In addition, fecal samples from 253 poultry, 213 bovines, 105 calves (under 1 year of age), 99 sheep, 22 dogs, 17 goats and 2 cats were collected. Upon first screening in Ethiopia (preliminary identification), *Campylobacter* was presumptively present in 250 of the 1014 samples. After shipping to the lab of microbiology, Ghent University, Belgium, presumptive isolates from 34 samples could not be cultivated or identified by PCR. The remaining isolates from
***250-34=216?***
129 samples could be recultivated and resulted in 135 identifications as *Campylobacter* species by MALDI-TOF MS, and an additional 97 identifications via PCR in 87 samples for which re-cultivation was not possible. From these confirmed 216 positive samples, 200 had one type of *Campylobacter* species, and 16 had co-infection with both *C. jejuni* and *C. coli*, resulting in the

identification of 232 *Campylobacter* isolates. The majority of the isolates were *C. jejuni* (n = 194) followed by *C. coli* (n = 34). Additionally, four animal isolates were identified as *C. fetus* (n = 2) and unspecified *Campylobacter* spp. (n = 2).

**Table 1. *Campylobacter* species occurrence and identification in children, backyard farm animals, and companion animals**

| **Number of hosts** | ***Campylobacter* species occurrence** | | | | | | ***Campylobacter* species identification** | | | | |
|---|---|---|---|---|---|---|---|---|---|---|---|
| | *C. jejuni* | *C. coli* | *C. jejuni* + *C. coli* | *C. fetus* | Unspecified *Campylobacter* spp. | **Total** | *C. jejuni* | *C. coli* | *C. fetus* | Unspecified *Campylobacter* spp. | **Total** |
| | N (%) | N (%) | N (%) | N (%) | N (%) | N (%) | N (%) | N (%) | N (%) | N (%) | N (%) |
| Children (n = 303) | 57 (19) | 2 (1) | 0 | 0 | 0 | 59 (20) | 57 (19) | 2 (1) | 0 | 0 | 59 (20) |
| Poultry (n = 253) | 91 (36) | 11 (4) | 10 (4) | 0 | 0 | 112 (44) | 101 (40) | 21 (8) | 0 | 0 | 122 (48) |
| Bovines (n = 213) | 10 (5) | 1 (1) | 0 | 1 (1) | 0 | 12 (6) | 10 (5) | 1 (1) | 1 (1) | 0 | 12 (6) |
| Calves (n = 105) | 4 (4) | 2 (2) | 1 (1) | 1 (1) | 1 (1) | 9 (9) | 5 (5) | 3 (3) | 1 (1) | 1 (1) | 10 (10) |
| Sheep (n = 99) | 5 (5) | 1 (1) | 3 (3) | 0 | 0 | 9 (9) | 8 (8) | 4 (4) | 0 | 0 | 12 (12) |
| Dogs (n = 22) | 11 (50) | 1 (5) | 2 (9) | 0 | 0 | 14 (64) | 13 (59) | 3 (14) | 0 | 0 | 16 (73) |
| Goats (n = 17) | 0 | 0 | 0 | 0 | 1 (6) | 1 (6) | 0 | 0 | 0 | 1 (6) | 1 (6) |
| Cats (n = 2) | 0 | 0 | 0 | 0 | 0 | 0 | 0 | 0 | 0 | 0 | 0 |
| **Total (n = 1014)** | 178 (18) | 18 (2) | 16 (2) | 2 (0.2) | 2 (0.2) | 216 (22) | 194 (19) | 34 (3) | 2 (0.2) | 2 (0.2) | 232 (23) |

In total, 59 of the isolates originated from the stools of 59 children (out of 303 samples), and 173 isolates from 157 animal fecal samples (out of 711 samples). The occurrence of *Campylobacter* in animals varied per host, with rates up to 64% in dogs to 6% in bovines (Table 1). In animals, co-occurrence of *C. jejuni* and *C. coli* was observed in dogs (9%), poultry (4%), sheep (3%), and calves (1%) (Table 1).

The isolation of *C. jejuni* was highest in dogs (59%), followed by poultry (40%), and children (19%), with 8%, 5% and 5% rates in bovines, sheep, and calves, respectively. Likewise, *C. coli* was isolated most from dogs (14%), then from poultry (8%), sheep (4%), calves (3%), children (1%), and bovines (1%).

The occurrence of *Campylobacter* is shown in Fig 2. Of the 1014 samples examined, *C. jejuni* was present in 194 (19%) and *C. coli* in 34 (3%), with 16 out of 711 samples from animal origin only (2%), with co-occurrence of *C. jejuni* and *C. coli*. *Campylobacter fetus* was isolated from one bovine and one calf fecal sample. In total at least one *Campylobacter* species was present in 216 of the 1014 samples examined (21%).

**Demographic and clinical details of children with diarrhea and *Campylobacter* infection**

The median age of the 303 children was 19 months (IQR, 12 - 31.5), and children with *Campylobacter* infection were significantly younger (13 months) compared to those without (19 months) (Mann-Whitney U test: $p < 0.001$). Based on the age groups classification of the Ethiopian Public Health Institute [26], this could further be specified to a significant variation in the proportion of *Campylobacter* positive cases per age group ($p = 0.002$) (Table 2), and upon post hoc analysis, a significantly higher number of infections were identified in the age groups 6-11 months as compared to 12-23 months ($p = 0.05$), 36-47 months ($p = 0.006$) and 48-60 months ($p = 0.004$) in our cohort (Fig 3).

**Table 2. Demographic and clinical details, and occurrence of *Campylobacter* in children under five with diarrhea.**

| Characteristics | *Campylobacter* in the stool | | Total N (%) | P-value |
|---|---|---|---|---|
| | Present | Not present | | |
| | N (%) | N (%) | | |
| **Gender** | | | | |
| Girls | 25 (18) | 115 (82) | 140 (46) | |
| Boys | 34 (21) | 129 (79) | 163 (54) | 0.511 |
| **Age (months)** | | | | |
| < 6 | 2 (22) | 7 (78) | 9 (3) | |
| 6-11 | 21 (38) | 34 (62) | 55 (18) | 0.002 |
| 12-23 | 20 (17) | 101 (83) | 121 (40) | |
| 24-35 | 9 (17) | 44 (83) | 53 (18) | |
| 36-47 | 3 (9) | 32 (91) | 35 (12) | |
| 48-60 | 2 (7) | 26 (93) | 28 (9) | |
| **Median age (IQR)** | 13 (10-22.8) | 19 (13-33.75) | 19 (12 -31.5) | < 0.001 |
| **Frequency of stool per day** | | | | |
| 3 - 5 | 48 (16) | 200 (66) | 248 (82) | |
| 6 -10 | 11 (20) | 44 (80) | 55 (18) | 0.913 |
| **Median (IQR)** | 4 (3-5) | 4 (3-5) | 4 (3-5) | 0.538 |
| **Duration of diarrhea (days)** | | | | |
| 1 – 2 | 18 (16) | 97 (84) | 115 (38) | |
| 3 – 5 | 33 (24) | 106 (76) | 139 (46) | 0.224 |
| 6 – 7 | 8 (16) | 41 (84) | 49 (16) | |
| **Median (IQR)** | 3 (2-5) | 3 (2-4) | 3 (2-4) | 0.242 |
| **Watery diarrhea** | | | | |
| Yes | 40 (20) | 158 (79) | 198 (65) | 0.659 |
| No | 19 (18) | 86 (82) | 105 (35) | |
| **Foul smelling diarrhea** | | | | |
| Yes | 37 (20) | 148 (80) | 185 (61) | 0.728 |
| No | 22 (19) | 96 (81) | 118 (39) | |
| **Bloody stools** | | | | |
| Yes | 4 (14) | 25 (86) | 29 (10) | 0.417 |
| No | 55 (20) | 219 (72) | 274 (90) | |
| **Vomiting** | | | | |
| Yes | 26 (17) | 124 (83) | 150 (50) | 0.352 |
| No | 33 (22) | 120 (78) | 153 (51) | |
| **Abdominal pain** | | | | |
| Yes | 36 (22) | 130 (78) | 166 (55) | 0.284 |
| No | 23 (17) | 114 (83) | 137 (45) | |
| **Fever** | | | | |
| Yes | 22 (21) | 82 (79) | 104 (34) | 0.593 |
| No | 37 (19) | 162 (81) | 199 (66) | |

IQR, interquartile range; Q1-Q3, lower quartile-upper quartile

(2+21+20+9+3+2=57)?? where are the two?

The summation is 301 too!!

How is calculated?

In our cohort, a total of 163 children (54%) were boys, and 140 (46%) were girls. A non-significant higher occurrence of *Campylobacter* was observed in boys (n = 34; 21%) as compared to girls (n = 25; 18%) (Table 2). The children had on average two siblings regardless of their diarrheic status and no associations between the number of siblings and *Campylobacter* isolation was found. The duration of diarrhea upon sampling was 3 to 5 days in 46% of children participating. The median duration of diarrhea at presentation was 3 days in both children with *Campylobacter* in their stool and those without. Likewise, the median stool frequency at presentation was 4 times per day in both groups. Watery diarrhea, abdominal pain, vomiting, and bloody stool were reported in 65%, 55%, 50%, and 10% of the children, respectively, with no significant association to *Campylobacter* infection (Table 2).

### Occurrence of *Campylobacter* at the household level

Out of 303 households, 245 households could be visited for the subsequent collection of 711 additional animal fecal samples. The median number of animals included per household was 3 (IQR, 2-4). *Campylobacter* was present in at least one host in 149 (61%) of 245 households. In 27 (18%) of these, *Campylobacter* was only detected in the child, while in 23 (15%) it was detected in both a child and an animal species (Fig 4A). *Campylobacter* was detected in a child and poultry in 14 of these 23 households, as well as in child-bovine, child-dog-poultry, child-sheep, child-bovine-dog, and child-calf-poultry combinations in 2, 2, 1, 1, and 1 households, respectively (Fig 4B). The majority (19/23) of these co-occurring identifications were consistent at *Campylobacter* species level between the different hosts. More specifically, 18 were consistent between hosts and were identified as *C. jejuni*, and one identified as *C. coli*. In four cases, different species of *Campylobacter* were identified in different hosts (child and animals) from the same household (Fig 4B).

*Campylobacter* was detected in animal species but not in children in 99 households. Among these, 89 were detected in a single animal species, with 68 (76%) from poultry, 7 (8%) from calves, 5 (6%) from dogs, 5 (6%) from sheep, 3 (3%) from bovines, and 1 (1%) from goats (Fig 4C). *C. jejuni* was responsible for 65 (73%), *C. coli* for 9 (10%), both for 11 (12%), *C. fetus* for 2 (2%), and unidentified *Campylobacter* spp. for 2 (2%) of identifications in the 89 households (Fig 4C). In 10 households, *Campylobacter* occurred in multiple animal species: 4 involved poultry and bovine, 3 poultry and dogs, 1 poultry and sheep, 1 poultry and calf, and 1 bovine and dog. Of these, *C. jejuni* was identified in all hosts in seven households, while in three households a variety of

*Campylobacter* species was identified (Fig 4C). Co-occurrence of both *C. jejuni* and *C. coli* in one host was observed exclusively in animal hosts in 9% (14/149) of households and mainly occurred in poultry (8 cases; 57%).

**Antibiotic resistance profiles in *Campylobacter***

As shown in Table 3, MIC values were read and interpreted for 128 *Campylobacter* isolates (103 *C. jejuni*, 24 *C. coli*, and one *C. fetus*). Isolates demonstrated resistance rates of 20% and 11% to ciprofloxacin and doxycycline, respectively. *C. coli* had higher ciprofloxacin resistance (50%) than *C. jejuni* (14%) ($p < 0.001$). Resistance to doxycycline was higher in *C. coli* (21%) compared to *C. jejuni* (9%) ($p = 0.088$).

**Table 3. Antibiotic resistance profiles of 103 *C. jejuni,* 24 *C. coli* and one *C. fetus,* isolated from children and animals.**

| **Isolates per source** | **Azithromycin** | **Erythromycin** | **Ciprofloxacin** | **Doxycycline** |
|---|---|---|---|---|
| | | | | |
| | N (% R) | N (% R) | N (% R) | N (% R) |
| **Children** | | | | |
| *C. jejuni* (n = 35) | 1 (3) | 0 | 3 (9) | 2 (6) |
| *C. coli* (n = 1) | 0 | 0 | 0 | 0 |
| **Poultry** | | | | |
| *C. jejuni* (n = 54) | 0 | 1 (2) | 10 (19) | 7 (13) |
| *C. coli* (n =19) | 0 | 0 | 9 (47) | 5 (26) |
| **Dogs**<br>*C. jejuni* (n = 7) | 0 | 0 | 1 (14) | 0 |
| **Ruminants** | | | | |
| *C. jejuni* (n = 7) | 0 | 0 | 0 | 0 |
| *C. coli* (n = 4) | 0 | 0 | 3 (75) | 0 |
| *C. fetus* (n = 1) | 0 | 0 | 0 | 0 |
| **Total *C. jejuni*** (n = 103) | 1 (1) | 1 (1) | 14 (14) | 9 (9) |
| **Total *C. coli*** (n = 24) | 0 | 0 | 12 (50) | 5 (21) |
| **Overall (n = 128)** | **1 (1)** | **1 (1)** | **26 (20)** | **14 (11)** |

n, number of resistant isolates; % R, percentage of resistance

The proportion of *C. jejuni* isolates resistant to ciprofloxacin was 3 (9%) in children and 10 (19%) in poultry. The proportion of *C. coli* isolates resistant to ciprofloxacin was 3 (75%) in ruminants and 9 (47%) in poultry. All *C. coli* isolates (n = 5) resistant to doxycycline were from poultry. Of the 128 isolates tested, only 1 (1%) from a child was resistant to azithromycin and 1 (1%) from poultry to erythromycin (Table 3).

Overall, (10/128, 8%) co-resistance to ciprofloxacin and doxycycline was obtained. The proportion of co-resistance was significantly higher in *C. coli* (n = 5, 21%) than in *C. jejuni* (n = 6, 6%) (p = 0.019). The proportion of co-resistance to ciprofloxacin and doxycycline was (n = 5, 5%) and (n = 5, 21%) in *C. jejuni* and *C. coli* isolates, respectively. All *C. coli* isolates and the majority of *C. jejuni* isolates resistant to both ciprofloxacin and doxycycline were observed in poultry.

The MICs of ciprofloxacin ranged from 0.008 to > 32 mg/L in *C. coli* and from 0.002 to > 32 mg/L in *C. jejuni*. The MICs of doxycycline ranged from 0.03 to 64 mg/L in *C. coli*, and from 0.016 to 256 mg/L in *C. jejuni*. Ciprofloxacin has a bimodal distribution of MICs for *C. coli* isolates (0.008-0.125 mg/L versus 4-32 mg/L). The MICs for doxycycline are also bimodal (Table 4).

**Table 4. Distribution of antimicrobial susceptibility results to four antibiotics of 103 *C. jejuni* and 24 *C. coli*, isolated from children with diarrhea and farm animals.**

| | | **Number of isolates with the following MIC (mg/L)** | | | | | | | | | | | | | | | | | |
|---|---|---|---|---|---|---|---|---|---|---|---|---|---|---|---|---|---|---|---|
| | **Clinical breakpoint for resistance (mg/L)** | **0.002** | **0004** | **0.008** | **0. 016** | **0. 03** | **0.06** | **0.125** | **0.25** | **0.5** | **1** | **2** | **4** | **8** | **16** | **32** | **48** | **64** | **256** |
| Azithromycin | | | | | | | | | | | | | | | | | | | |
| *C. jejuni* | > 4 | | | | 12 | 37 | 42 | 9 | | 1 | | | 1 | | | | | | 1 |
| *C. coli* | > 8 | | | | | 2 | 3 | 8 | 6 | 5 | | | | | | | | | |
| Ciprofloxacin | | | | | | | | | | | | | | | | | | | |
| *C. jejuni* | > 0.5 | 3 | 3 | 14 | 22 | 26 | 17 | 2 | 2 | | | 1 | | 1 | | 12 | | | |
| *C. coli* | > 0.5 | | | 1 | 1 | 5 | 4 | 1 | | | | | 1 | 1 | | 10 | | | |
| Doxycycline | | | | | | | | | | | | | | | | | | | |
| *C. jejuni* | > 2 | | | | 52 | 33 | 2 | 5 | 1 | | | 1 | | 3 | 3 | | | 1 | 2 |
| *C. coli* | > 2 | | | | | 16 | 3 | | | | | | | | | 1 | | 4 | |
| Erythromycin | | | | | | | | | | | | | | | | | | | |
| *C. jejuni* | > 4 | | | | 3 | 14 | 28 | 24 | 17 | 11 | 3 | 1 | 1 | | | | | 1 | |
| *C. coli* | > 8 | | | | | | | 2 | 5 | 8 | | 9 | | | | | | | |

MIC, minimum inhibitory concentration

The grey shading indicates MIC values of the resistant isolates

As shown in Table 5, of the *C. jejuni* isolates simultaneously isolated from a child and poultry in 4 households, all were concurrently susceptible to azithromycin, doxycycline and erythromycin. However, in one household, a poultry isolate was resistant to doxycycline, and in another household, a poultry isolate was resistant to erythromycin. For ciprofloxacin, two isolates were concurrently susceptible, increased exposure, and two were resistant in poultry but not in children.

In two households where *C. jejuni* was isolated from dogs and poultry, isolates from both sources were susceptible to erythromycin and azithromycin. However, in one household, an isolate from a poultry was resistant to ciprofloxacin, and in another household, a poultry isolate was resistant to doxycycline (Table 5). Furthermore, from three households, where *C. jejuni* and *C. coli* were isolated from a single poultry sample, both species were susceptible to all antibiotics. Similarly, from a single calf in a household, both *C. jejuni* and *C. coli* isolated were susceptible to all antibiotics (Table 5).

**Table 5. Antibiotic susceptibility profiles of concurrent *Campylobacter* species isolated from the child and animal species, and from distinct animal species per homes.**

| ***Campylobacter* Positive home number** | **Hosts per home** | ***Campylobacter* species** | **Antibiotics tested** | | | |
|---|---|---|---|---|---|---|
| | | | **Erythromycin** | **Ciprofloxacin** | **Azithromycin** | **Doxycycline** |
| | **A child and animal species** | | | | | |
| 90 | Child | *C. jejuni* | S | I | S | S |
| | Poultry | *C. jejuni* | R | R | S | S |
| 139 | Child | *C. jejuni* | S | I | S | S |
| | Poultry | *C. jejuni* | S | I | S | S |
| 239 | Child | *C. jejuni* | S | I | S | S |
| | Poultry | *C. jejuni* | S | R | S | S |
| 256 | Child | *C. jejuni* | S | I | S | S |
| | Dog | *C. jejuni* | S | I | S | S |
| 288 | Child | *C. jejuni* | S | I | S | S |
| | Poultry | *C. jejuni* | S | I | S | R |
| | **Different animal species** | | | | | |
| 117 | Dog | *C. jejuni* | S | I | S | S |
| | Poultry | *C. jejuni* | S | R | S | S |
| 280 | Dog1 | *C. jejuni* | S | I | S | S |
| | Dog2 | *C. jejuni* | S | I | S | S |
| | Poultry1 | *C. jejuni* | S | I | S | R |
| | Poultry2 | *C. jejuni* | S | I | S | S |
| | **A single animal species** | | | | | |
| 108 | Poultry | *C. coli* | S | I | S | S |
| | | *C. jejuni* | S | I | S | S |
| 124 | Poultry | *C. jejuni* | S | I | S | S |
| | | *C. coli* | S | I | S | S |
| 196 | Calf | *C. coli* | S | I | S | S |
| | | *C. jejuni* | S | I | S | S |
| 205 | Poultry | *C. coli* | S | I | S | S |
| | | *C. jejuni* | S | I | S | S |

S, Susceptible; R, Resistant: I, Susceptible, increased exposure

## Discussion

This study presents the occurrence, spatial distribution, and antibiotic resistance of *Campylobacter* in a large cohort of under five children with acute diarrhea and various animal species. Using a membrane filtration culture technique and a conventional microaerobic incubation system, we successfully isolated thermotolerant *Campylobacter*. MALDI-TOF MS and PCR confirmed 86% of presumptive isolates as *Campylobacter*. Compared to earlier regional studies *(???)*, the rate of infection in children is high, with children aged 6-11 months having the highest infection rates. Occurrences at household-level suggest direct animal-to-child transmission. Antibiotic resistance profiles showed low resistance to erythromycin and azithromycin but higher rates for ciprofloxacin and doxycycline, particularly in poultry. The findings could be applicable to regions with similar socio-economic and cultural contexts, where backyard farming is common.

The fastidious nature of *Campylobacter* challenges the reliability of stool culture for estimation of the burden of *Campylobacter* in regions with ~~no specialist~~ **restricted resources for** laboratories. Previous studies in Ethiopia lacked consistency in culture techniques impeding a comprehensive understanding of *Campylobacter* epidemiology. To address this gap, the current study, the first of its kind in Ethiopia, adopted a membrane filtration culture technique that resulted in the successful isolation of thermotolerant *Campylobacter* from children, poultry and dogs **farm animals and pet animals**. A significant portion of presumptive samples were confirmed as *Campylobacter* via molecular tools.

Out of the 232 *Campylobacter* identifications, 58% were identified using MALDI-TOF MS, and 42% via PCR. The cells which could not be resuscitated and recovered by culture but that were identified via PCR, could be the viable but non-culturable (VBNC) cells [42], or injured cells due to freeze-thaw during an unstable power supply [43], or the lack of representation in the MALDI-TOF MS database. Moreover, presumptive isolates in a subset of samples not identified as *Campylobacter* by molecular tools could be bacteria such as *Proteus* and *Acinetobacter*, which resemble *Campylobacter* morphology on mCCDA media [44], or emerging *Campylobacter* species.

Compared to earlier studies in different regions of Ethiopia, the rate of *Campylobacter* infection in children (20%) in the present study, exceeded the rates reported in southwest (9-17%) [45, 46], southern (7-13%) [47, 48], and northwest (6-15%) [49-51] Ethiopia. These studies typically used

selective *Campylobacter* agar without filtration, and incubation at 42°C for 24 to 48 hours. A study in eastern Ethiopia reported a detection rate of 15% in infants by PCR [7]. However, another study from southern Ethiopia [25], reported detection rate of 49% in infants using selective chromogenic agars. The rate of infection in children is also higher than previously reported rates in Africa [9], Asian countries [52-54], Ecuador [55] and Poland [56]. The variation in the findings could be due to the stool culture techniques used to isolate and identify *Campylobacter*. The studies in Africa [9, 45, 46, 48, 50, 51] applied biochemical tests that have identification limitations stemming from the low metabolic activity of *Campylobacter* [34]. Thus, the membrane filtration culture method, combined with specific incubation conditions is an effective and easy method for screening *C. jejuni*/*C. coli* in routine diagnosis, and research in settings lacking organized laboratories.

In the present study, the number of *Campylobacter* infection were significantly higher in children ?? between the ages of 6 and 11 months compared to the two oldest age groups, in line with previous studies in Ethiopia [51], and elsewhere in the world [57-61]. As reported in Ethiopia [45, 46, 51] and Uganda [62], higher infection rates were observed in children below three years old in the present study. This can be attributed to differences in the development and maturation of intestinal microbiota among the different age groups [63]. From the age of two years onwards, a more matured intestinal microbiota has been reported in children [64, 65]. Besides, children between 6 and 11 months are more likely to be exposed to contaminated surfaces, foods, and water after weaning, and have less developed immunity [9, 63].

**Any stat. test?**

How about earlier exposure to campy induce specific 'partially' protective immunity?

In our  study, *C. jejuni* prevailed *C. coli* in both children and animal species consistent with what is reported before [22]. This could be due to the diverse nature of *C. jejuni*, enabling its widespread colonization in mammals and poultry [43]. Moreover, *C. jejuni* has better virulence factors and adaptation mechanism that enable it to survive and proliferate in the human gastrointestinal tract compared to *C. coli* [43, 66]. The observed occurrence of *C. jejuni* in children (19%) in the present study exceeded previously documented rates in Ethiopia (9 to 12%) [46, 67] as well as rates reported among children in the African context (5.2-12.3%) [9]. The rate of *C. jejuni* in ruminants (5-8%) is comparable with previous reports from other parts of Ethiopia (ranging from 3% to 9%) [24, 68, 69]. A higher rate of *C. jejuni* has already been reported from the feces of poultry (81%) and cattle (54%) in southwest Ethiopia [70]. Methodological differences could contribute to the observed variations, as several earlier studies in Ethiopia relied on biochemical tests [47, 68-70].

The overall rate of *C. coli* (3%) in the present study fell within the spectrum of rates (ranging from 1% to 11%) reported in previous studies mentioned above [23, 46, 67, 68, 70]. The isolation rate of *Campylobacter coli* was higher in animal species than children in the present study. The strains of *C. coli* prevalent in animals might be less adapted to infect and cause disease in children. Additionally, the simultaneous excretion of both *C. jejuni* and *C. coli* was observed in backyard farm animals and companion animals but not in children. This could be due to competition with the intestinal microbiota, and variations in exposure to pathogens, body temperature, strain virulence, and immune responses [29, 71].

Apart from a study in central Ethiopia reporting an 8% prevalence of *C. fetus* from livestock [23], other studies in Ethiopia did not isolate *C. fetus* from fecal animal samples. The use of an incubation temperature of 42° C inhibits the growth of most non-*C. jejuni* and non-*C. coli* *Campylobacter* spp. The presence of *C. fetus* only in one bovine and one calf stool sample and the inability to isolate other slow-growing *Campylobacter* species in the present study might be linked to the use of microaerobic conditions without hydrogen during isolation [31, 72, 73].

Regarding animal species, the occurrence of *Campylobacter* spp. was higher in dogs and poultry compared to sheep and cattle in concurrence with a finding from southwest Ethiopia where the percentage of isolation was higher in chicken compared to sheep and cattle [70]. Conversely, other studies in central and southwest region of Ethiopia reported higher rates of *C. jejuni* in ruminants and *C. coli* in sheep [23, 70, 74]. The isolation rate of *Campylobacter* among household dogs (64%) was higher compared to rates from other countries (11-45.4%) [75, 76], possibly due to the unrestricted movements of dogs in the neighborhood, potentially increasing their exposure to various sources of contamination, including the excreta of children and from consumption of dairy products [77]. Similarly, the isolation rate of *Campylobacter* in poultry (44%) was higher than a report from central Ethiopia (13%) [23]. Relatively lower rates of *Campylobacter* isolation in sheep (9%) and cattle (6-9%) were documented compared to previous reports in Ethiopia (11%-38% in sheep and 12.6-12.7% in cattle) [20, 69, 70]. However, considering the fact that 85% of households in Ethiopia own backyard farm animals [26], and keep them in families house, and the concurrent excretion of *C. jejuni* and *C. coli* in a few cattle in the current study highlights the need for further research. Differences in animal species, age, and handling practices and geographic

regions may be attributed to the observed differences in *Campylobacter* occurrences among backyard farm animals.

As a pathogen of threat to One Health, assessing *Campylobacter* transmission between children and animals sharing the same house is crucial for designing targeted interventions [78]. *Campylobacter* was detected in 61% of the family's homes visited, which is higher than a previous study from central Ethiopia (42%) [23]. This could be due to variations in the age and diarrhea status of human participants. We screened stool samples collected only from children under five with diarrhea at health centers, while the study in central Ethiopia collected pooled human fecal samples at the household level regardless of both diarrheal status and age specifications.

Regarding transmission dynamics within the houses, the occurrence of joint cases of *C. jejuni* among children and animals (for instance, child-poultry in 14 houses, child-bovines in 2 and child-dog-poultry in 2 homes) suggest direct transmission of *Campylobacter* from animals to children via exposure to floors and various surfaces contaminated with the feces of animals. This is strengthened by the free access of animals to sitting, kitchen, and ~~eating~~ dining areas of the families as observed during data collection. This finding is in accordance with other studies [23, 79, 80]. However, the detection of *Campylobacter* exclusively in children in some homes or exclusively in animals in other homes could indicate different contamination sources [79].

Knowledge on the antibiotic resistance of *Campylobacter* isolates from children, backyard farm animals, and companion animals is scarce in Ethiopia. The present study determined, for the first time in Ethiopia, MICs for the antibiotics recommended to treat human campylobacteriosis. Only 0.8% of tested *C. jejuni* isolates were resistant to erythromycin and azithromycin, which is consistent with recent CDC, European Union (EU), and global reports [56, 81-83]. However, a MIC of azithromycin ($\geq$ 256 mg/l) was observed in a *C. jejuni* from a child. This could be due to the unrestrained unrestricted use of azithromycin for mild pneumonia in the study area [84]. Higher rates of resistance to erythromycin were reported in *Campylobacter* isolates from young children in southwest Ethiopia (11-18%) [45, 46], and northwest Ethiopia (28%) [50] and in poultry in central Ethiopia (15%) [23]. These studies employed disc diffusion techniques for susceptibility testing.

The 20% rate of ciprofloxacin resistance is consistent with findings in Europe (18-22%) [71, 85]. However, variable resistance rates to ciprofloxacin were reported in isolates from children in

southwest (5.3% to 15.8%) [45, 46], southern (55%) [47] and northwest (16%) [50] Ethiopia. An 8% rate of resistance in bovines and 13% combined (human and backyard farm animals) were also reported in Ethiopia [20, 23]. *C. coli* displayed significantly higher resistance to ciprofloxacin than *C. jejuni* ($p < 0.001$), consistent with reports from EU [82] and other country [83]. The high rates of ciprofloxacin resistance among *C. jejuni* isolates from poultry and *C. coli* isolates from calves (67%) in the present study is concurrent with a study from Poland for *C. jejuni* [86]. and a report from EU countries for *C. coli* [81]. As only a few isolates from ruminants were tested in this study, caution needs to be taken in the interpretation of this finding. The MIC of ciprofloxacin showed a bimodal distribution in *C. coli* compared to a monomodal one in *C. jejuni* indicating different resistance mechanisms or the presence of subpopulations in *C. coli*. The MICs for doxycycline are also bimodal and in agreement with the EUCAST epidemiological cut-offs (ECOFFs).

The overall rate of *Campylobacter* resistance to doxycycline (11%) was lower compared to worldwide reports (28-42%) [87, 88]. The rate of resistance was considerably higher in *C. coli* than in *C. jejuni*, consistent with reports from the EU [81]. The rate of doxycycline resistance in *C. jejuni* was higher among isolates from poultry than children consistent with the study from Poland [86]. The 8% ciprofloxacin-doxycycline cross-resistance observed in *Campylobacter* isolates in the present study is in line with the 6% and 9% rates reported in U.S., [11] but lower than the 15% cross-resistance rates in Bulgaria [85]. Almost 90% of ciprofloxacin-doxycycline cross-resistance was observed among isolates from poultry. The overall cross-resistance was significantly higher in *C. coli* isolates than *C. jejuni*, consistent with the report from EU [81].

This study presents the matching between susceptibility profiles of isolates from children and animals (Table 5). Presence of matched AST profiles in some homes was confirmed, while unmatched in others. In general, the resistance of *Campylobacter* to ciprofloxacin and doxycycline in the present study may be due to the common use of these antibiotics for treating enteric infections in the study area. The predominance of resistance in isolates from backyard poultry where *Campylobacter* can easily multiply, survive in droppings for up to 6 days after excretion [89] and spread to children, humans, and companion animals is of particular concern. The observed resistance in poultry may be due to specific poultry-associated mechanisms, reverse zoonotic transmission, acquired resistance from wild birds, local farming, or environmental sources, rather than selection pressure, as antibiotics are not used in backyard poultry in this particular setting.

Moreover, the unmatched AST profiles of *C. jejuni* isolates across poultry and children, and poultry and dogs observed in a few homes (Table 5) support these explanations.

Assessment of the occurrence of *Campylobacter* using large sample sizes at the children-animal interface, the use of membrane filtration culture technique for the first time in Ethiopia, as well as successful transportation from Ethiopia to Belgium on dry ice, and subsequent recultivation under microaerobic conditions by introducing a gas mixture, were the strengths of this study. In addition, the confirmation of isolates using MALDI-TOF MS and determination of ABR based on gradient strip diffusion susceptibility testing is the first for *Campylobacter* from Ethiopia. However, the microaerobic condition without a hydrogen-enriched atmosphere used limited the isolation of *Campylobacter* species other than *C. jejuni* and *C. coli*.

In conclusion, *C. jejuni* is prevalent in children below the age of 3 years with diarrhea in Ethiopia. Its abundance in children, shedding from poultry and companion animals, and concordant presence in both a child and animal species with matching AST profiles in certain homes suggest direct infection from animals rather than through food. Based on the finding, treatment of severe campylobacteriosis in children should be guided by E-test susceptibility testing. In order to preserve efficacy and minimize the risk of antibiotic resistance development, azithromycin and erythromycin should only be used for the treatment of severe campylobacteriosis that is resistant to ciprofloxacin. The observed high prevalence of *Campylobacter* in children and the potential for direct transmission from animals to children within households suggest further genomic analysis to comprehensively understand *Campylobacter* transmission and the mechanism of ABR, and its burden on One Health. This finding highlights the need for targeted interventions such as raising awareness of transmission routes, improving hygiene, and proper handling of animals and disposal of their waste. Clinicians, veterinarians, and policymakers should integrate these findings for evidence-based diagnosis and treatment, further research, surveillance, and mitigation of *Campylobacter* transmission.

**Acknowledgments**

Special thanks to Ghent University for the fellowship and LM-UGent for hosting the laboratory analysis. We are indebted to Bahir Dar University for financial support for data collection and laboratory consumables in Ethiopia. Special thanks to Margo Cnockaert, Martine Boonaert and Carine van Lancker for providing training and excellent technical assistance. The authors gratefully acknowledge the technical skills and support of the technicians of the microbiology laboratory in Saint-Lucas Hospital. We are very grateful to the children and their parents who participated in the study. Thanks to local health administrations for their crucial support and facilitation of the field work during the Covid-19 pandemic and wartime in Ethiopia. The authors would like to thank to the Microbiology staff of the Amhara Public Health Institute and Felegehiwot Referral Hospital for providing sterile sheep blood. We extend our thanks to healthcare staffs of the study health centers for their assistance in data and stool sample collection. We thank community workers, and volunteers for their unreserved help and coordination of the field work. Special thanks to Mr. Aynenew Abate and Mr. Belaynew Kerisew, and the local motorcyclists for their community awareness and support in the house to house data collection. We thank Dr. Ashebir Sewale Belay for mapping the study area and spatial distribution of health centers and the houses.

**Author Contributions**

**Conceptualization**: Wondemagegn Mulu, Marie Joossens, Mulugeta Kibret, Kurt Houf
**Data curation**: Wondemagegn Mulu
**Formal analysis**: Wondemagegn Mulu, Marie Joossens
**Funding acquisition**: Wondemagegn Mulu, Marie Joossens, Mulugeta Kibret, Kurt Houf
**Investigation**: Wondemagegn Mulu, Marie Joossens, Anne-Marie Van den Abeele, Kurt Houf
**Methodology**: Wondemagegn Mulu, Marie Joossens, Mulugeta Kibret, Anne-Marie Van den Abeele, Kurt Houf
**Project administration**: Wondemagegn Mulu, Marie Joossens, Mulugeta Kibret, Kurt Houf
**Resources**: Mulugeta Kibret, Marie Joossens, Kurt Houf
**Supervision**: Marie Joossens, Mulugeta Kibret, Anne-Marie Van den Abeele, Kurt Houf
**Validation**: Wondemagegn Mulu, Kurt Houf

**Visualization**: Wondemagegn Mulu, Marie Joossens, Anne-Marie Van den Abeele, Kurt Houf

**Writing- Original draft preparation**: Wondemagegn Mulu, Marie Joossens, Kurt Houf

**Writing-Review and editing**: Wondemagegn Mulu, Marie Joossens, Mulugeta Kibret, Anne-Marie Van den Abeele and Kurt Houf

## References

1. Hartman RM, Cohen AL, Antoni S, Mwenda J, Weldegebriel G, Biey J, et al. Risk factors for mortality among children younger than age 5 years with severe diarrhea in Low-and Middle-income Countries: Findings From the World Health Organization-coordinated Global Rotavirus and Pediatric Diarrhea Surveillance Networks. Clin Infect Dis. 2023;76(3):e1047-e53.

2. Chukwu MO, Abia ALK, Ubomba-Jaswa E, Dewar JB, Obi C. Mixed aetiology of diarrhoea in infants attending clinics in the North-West Province of South Africa: potential for sub-optimal treatment. Pathog. 2020; 9(3):198.

3. Samie A, Moropeng RC, Tanih NF, Dillingham R, Guerrant R, Bessong PO. Epidemiology of *Campylobacter* infections among children of 0–24 months of age in South Africa. Arch Public Health. 2022;80(1):107.

4. Lloyd-Evans N, Drasar B, Tomkins A. A comparison of the prevalence of *Campylobacter*, Shigellae and Salmonellae in faeces of malnourished and well nourished children in the Gambia and Northern Nigeria. Trans R Soc Trop Med Hyg. 1983;77(2):245-7.

5. Zenebe T, Zegeye N, Eguale T. Prevalence of *Campylobacter* species in human, animal and food of animal origin and their antimicrobial susceptibility in Ethiopia: a systematic review and meta-analysis. Ann Clin Microbiol Antimicrob. 2020;19(1):1-11.

6. Diriba K, Awulachew E, Anja A. Prevalence and associated factor of *Campylobacter* species among less than 5-year-old children in Ethiopia: a systematic review and meta-analysis. Eur J Med Res. 2021;26(1):1-10.

7. Terefe Y, Deblais L, Ghanem M, Helmy YA, Mummed B, Chen D, et al. Co-occurrence of *Campylobacter* species in children from Eastern Ethiopia, and their association with environmental enteric dysfunction, diarrhea, and host microbiome. Front Public Health. 2020;8:99.

8. Haque MA, Platts-Mills JA, Mduma E, Bodhidatta L, Bessong P, Shakoor S, et al. Determinants of *Campylobacter* infection and association with growth and enteric inflammation in children under 2 years of age in low-resource settings. Sci Rep. 2019;9(1):17124.

9. Asuming-Bediako N, Parry-Hanson Kunadu A, Abraham S, Habib I. *Campylobacter* at the human–food interface: the african perspective. Pathog. 2019;8(2):87.

10. Ghssein G, Awada R, Salami A, Bahmad HF, Awad A, Joumaa WH, et al. Prevalence, laboratory findings and clinical characteristics of campylobacteriosis agents among hospitalized children with acute gastroenteritis in Lebanon. Pediatr Gastroenterol Hepatol Nutr. 2021;24(4):346.

11. Metreveli M, Bulia S, Shalamberidze I, Tevzadze L, Tsanava S, Goenaga JC, et al. Campylobacteriosis, Shigellosis and Salmonellosis in hospitalized children with acute inflammatory diarrhea in Georgia. Pathog. 2022;11(2):232.

12. The WHO AWaRe (Access, Watch, Reserve) antibiotic book. Geneva: World Health Organization; 2022

13. Beyene AM, Andualem T, Dagnaw GG, Getahun M, LeJeune J, Ferreira JP. Situational analysis of antimicrobial resistance, laboratory capacities, surveillance systems and containment activities in Ethiopia: A new and One Health approach. One Health. 2023:100527.

14. Isada M, Reist M, MacKinnon M, Uhland F, Young K, Gibbens K, et al. Characterization of burden of illness measures associated with human (Fluoro) quinolone-resistant *Campylobacter* spp. infections-A scoping review. Epidemiol Infect. 2022:1-37.

15. da Silva Quetz J, Lima IFN, Havt A, de Carvalho EB, Lima NL, Soares AM, et al. *Campylobacter jejuni* and *Campylobacter coli* in children from communities in Northeastern Brazil: molecular detection and relation to nutritional status. Diagn Microbiol Infect Dis. 2010;67(3):220-7.

16. Houf K, De Smet S, Baré J, Daminet S. Dogs as carriers of the emerging pathogen Arcobacter. Vet Microbiol. 2008;130(1-2):208-13.

17. Sanchez JJ, Alam MA, Stride CB, Haque MA, Das S, Mahfuz M, et al. *Campylobacter* infection and household factors are associated with childhood growth in urban Bangladesh: An analysis of the MAL-ED study. PLoS Negl Trop Dis. 2020;14(5):e0008328.

18. On SL, Miller WG, Houf K, Fox JG, Vandamme P. Minimal standards for describing new species belonging to the families Campylobacteraceae and Helicobacteraceae: *Campylobacter*, *Arcobacter*, *Helicobacter* and *Wolinella* spp. Int J Syst Evol Microbiol. 2017;67(12):5296.

19. Same RG, Tamma PD. *Campylobacter* infections in children. Pediatr Rev. 2018;39(11):533-41.

20. Debelo M, Mohammed N, Tiruneh A, Tolosa T. Isolation, identification and antibiotic resistance profile of thermophilic *Campylobacter* species from Bovine, Knives and personnel at Jimma Town Abattoir, Ethiopia. PLoS One. 2022;17(10):e0276625.

21. Kelley BR, Ellis JC, Hyatt D, Jacobson D, Johnson J. Isolation and Whole-Genome Sequencing of Environmental *Campylobacter*. Curr Protoc Microbiol. 2018;51(1):e64.

22. Zhang Q, Beyi AF, Yin Y. Zoonotic and antibiotic-resistant *Campylobacter*: A view through the One Health lens. One Health Adv. 2023;1(1):4.

23. Chala G, Eguale T, Abunna F, Asrat D, Stringer A. Identification and characterization of *Campylobacter* species in livestock, humans, and water in livestock owning households of Peri-urban Addis Ababa, Ethiopia: A one health approach. Front Public Health. 2021;9:750551.

~~24. Terefe Y, Deblais L, Ghanem M, Helmy YA, Mummed B, Chen D, et al. Co-occurrence of *Campylobacter* species in children from Eastern Ethiopia, and their association with environmental enteric dysfunction, diarrhea, and host microbiome. Front Public Health. 2020;8:99.~~

25. Budge S, Barnett M, Hutchings P, Parker A, Tyrrel S, Hassard F, et al. Risk factors and transmission pathways associated with infant *Campylobacter* spp. prevalence and malnutrition: a formative study in rural Ethiopia. PLoS One. 2020;15(5):e0232541.

26. Ethiopian Public Health Institute (EPHI) and ICF. Ethiopia Mini Demographic and Health Survey 2019: Key Indicators. 2019.

27. Federal Democratic Republic of Ethiopia, Central Statistical Agency. Population projection of Ethiopia for all regions at Wereda level from 2014–2017. Addis Ababa; August 2013.

28. Cho Y-i, Yoon K-J. An overview of calf diarrhea-infectious etiology, diagnosis, and intervention. J Vet Sci. 2014;15(1):1-17.

29. Lazou T, Dovas C, Houf K, Soultos N, Iossifidou E. Diversity of *Campylobacter* in retail meat and liver of lambs and goat kids. Foodborne Pathog Dis. 2014;11(4):320-8.

30. Vandenberg O, Houf K, Douat N, Vlaes L, Retore P, Butzler J-P, et al. Antimicrobial susceptibility of clinical isolates of non-jejuni/coli campylobacters and arcobacters from Belgium. J Antimicrob Chemother. 2006;57(5):908-13.

31. Tilmanne A, Kandet Yattara HM, Herpol M, Vlaes L, Retore P, Quach C, et al. Multi-step optimization of the filtration method for the isolation of *Campylobacter* species from stool samples. Eur J Clin Microbiol. 2019;38:859-64.

32. Habib I, Miller WG, Uyttendaele M, Houf K, De Zutter L. Clonal population structure and antimicrobial resistance of *Campylobacter jejuni* in chicken meat from Belgium. Appl Environ Microbiol. 2009;75(13):4264-72.

33. Bhattarai V, Sharma S, Rijal KR, Banjara MR. Co-infection with *Campylobacter* and rotavirus in less than 5 year old children with acute gastroenteritis in Nepal during 2017–2018. BMC Pediatr. 2020;20(1):1-8.

34. Houf K, Stephan R. Isolation and characterization of the emerging foodborn pathogen Arcobacter from human stool. J Microbiol Methods. 2007;68(2):408-13.

35. Navarro-Gonzalez N, Ugarte-Ruiz M, Porrero M, Zamora L, Mentaberre G, Serrano E, et al. *Campylobacter* shared between free-ranging cattle and sympatric wild ungulates in a natural environment (NE Spain). Ecohealth. 2014;11:333-42.

36. Yu Z, Joossens M, Van den Abeele A-M, Kerkhof P-J, Houf K. Isolation, characterization and antibiotic resistance of *Proteus mirabilis* from Belgian broiler carcasses at retail and human stool. Food Microbiol. 2021;96:103724.

37. Yu Z, Joossens M, Kerkhof P-J, Houf K. Bacterial shifts on broiler carcasses at retail upon frozen storage. Int J Food Microbiol. 2021;340:109051.

38. Niemann S, Pühler A, Tichy HV, Simon R, Selbitschka W. Evaluation of the resolving power of three different DNA fingerprinting methods to discriminate among isolates of a natural Rhizobium meliloti population. J Appl Microbiol. 1997;82(4):477-84.

39. Eyers M, Chapelle S, Van Camp G, Goossens H, De Wachter R. Discrimination among thermophilic *Campylobacter* species by polymerase chain reaction amplification of 23S rRNA gene fragments. J Clin Microbiol. 1994;32(6):1623.

40. Denis M, Soumet C, Rivoal K, Ermel G, Blivet D, Salvat G, et al. Development of am-PCR assay for simultaneous identification of *Campylobacter jejuni* and *C. coli*. Lett Appl Microbiol. 1999;29(6):406-10.

41. EUCAST. The European Committee on Antimicrobial Susceptibility Testing. Clinical breakpoints. 2017.

42. Lv R, Wang K, Feng J, Heeney DD, Liu D, Lu X. Detection and quantification of viable but non-culturable *Campylobacter jejuni*. Front Microbiol. 2020;10:2920.

43. Houf K. *Campylobacter* and *Arcobacter*. 2020. Check for proper/complete citation of Houf, 2020!!

44. Sheludchenko M, Padovan A, Katouli M, Stratton H. *Acinetobacter baumannii* detected on modified charcoal cefoperazone deoxycholate agar in a waste stabilization pond. Can J Microbiol. 2020;66(3):206-13.

45. Nigusu Y, Abdissa A, Tesfaw G. *Campylobacter* gastroenteritis among under-five children in Southwest Ethiopia. Infect Drug Resist. 2022:2969-79.

46. Tafa B, Sewunet T, Tassew H, Asrat D. Isolation and antimicrobial susceptibility patterns of *Campylobacter* species among diarrheic children at Jimma, Ethiopia. Int J Bacteriol. 2014;2014.

47. Mulatu G, Beyene G, Zeynudin A. Prevalence of *Shigella*, Salmonella and *Cmpylobacter* species and their susceptibility patters among under five children with diarrhea in Hawassa town, South Ethiopia. Ethiop J Health Sci. 2014;24(2):101.

48. Behailu Y, Hussen S, Alemayehu T, Mengistu M, Fenta DA. Prevalence, determinants, and antimicrobial susceptibility patterns of *Campylobacter* infection among under-five children with

diarrhea at Governmental Hospitals in Hawassa city, Sidama, Ethiopia. A cross-sectional study. PLoS One. 2022;17(5):e0266976.

49. Mitike G, Kassu A, Genetu A, Nigussie D. Campylobacter enteritis among children in Dembia district, northwest Ethiopia. East Afr Med J. 2000;77(12).

50. Lengerh A, Moges F, Unakal C, Anagaw B. Prevalence, associated risk factors and antimicrobial susceptibility pattern of *Campylobacter* species among under five diarrheic children at Gondar University Hospital, Northwest Ethiopia. BMC Pediatr. 2013;13:1-9.

51. Gedlu E, Aseffa A. *Campylobacter* enteritis among children in north-west Ethiopia: a 1-year prospective study. Ann Trop Paediatr. 1996;16(3):207-12.

52. Ghssein G, Awada R, Salami A, Bahmad HF, Awad A, Joumaa WH, et al. Prevalence, laboratory findings and clinical characteristics of campylobacteriosis agents among hospitalized children with acute gastroenteritis in Lebanon. Pediatr Gastroenterol Hepatol Nutr. 2021;24(4):346.

53. Mohakud NK, Patra SD, Kumar S, Sahu PS, Misra N, Shrivastava AK. Detection and molecular typing of *Campylobacter* isolates from human and animal faeces in coastal belt of Odisha, India. Indian J Med Microbiol. 2019;37(3):345-50.

54. Rajendran P, Babji S, George A, Rajan D, Kang G, Ajjampur S. Detection and species identification of *Campylobacter* in stool samples of children and animals from Vellore, south India. Indian J Med Microbiol. 2012;30(1):85-8.

55. Vasco K, Graham JP, Trueba G. Detection of zoonotic enteropathogens in children and domestic animals in a semirural community in Ecuador. Appl Environ Microbiol. 2016;82(14):4218-24.

56. Szczepanska B, Andrzejewska M, Spica D, Klawe JJ. Prevalence and antimicrobial resistance of *Campylobacter jejuni* and *Campylobacter coli* isolated from children and environmental sources in urban and suburban areas. BMC Microbiol. 2017;17(1):1-9.

57. ~~Asuming-Bediako N, Parry-Hanson Kunadu A, Abraham S, Habib I. *Campylobacter* at the human–food interface: the african perspective. Pathog. 2019;8(2):87.~~

58. Rao MR, Naficy AB, Savarino SJ, Abu-Elyazeed R, Wierzba TF, Peruski LF, et al. Pathogenicity and convalescent excretion of *Campylobacter* in rural Egyptian children. Am J Epidemiol. 2001;154(2):166-73.

59. Cornejo-Tapia A, Orellana-Peralta F, Weilg P, Bazán-Mayra J, Cornejo-Pacherres H, Ulloa-Urizar G, et al. Etiology, epidemiology and clinical characteristics of acute diarrhea in hospitalized children in rural Peru. J Infect Dev Ctries. 2017;11(11):826-32.

60. Sadiq A, Bokhari H, Noreen Z, Asghar RM, Bostan N. Magnitude of Rotavirus A and *Campylobacter jejuni* infections in children with diarrhea in Twin cities of Rawalpindi and Islamabad, Pakistan. BMC Infect Dis. 2019;19:1-10.

61. Sakran W, Hexner-Erlichman Z, Spiegel R, Batheesh H, Halevy R, Koren A. *Campylobacter* gastroenteritis in children in north-eastern Israel comparison with other common pathogens. Sci Rep. 2020;10(1):5823.

62. Mshana S, Joloba M, Kakooza A, Kaddu-Mulindwa D. *Campylobacter* spp among Children with acute diarrhea attending Mulago hospital in Kampala-Uganda. Afr Health Sci. 2009;9(3):201.

63. Randremanana RV, Randrianirina F, Sabatier P, Rakotonirina HC, Randriamanantena A, Razanajatovo IM, et al. *Campylobacter* infection in a cohort of rural children in Moramanga, Madagascar. BMC Infect Dis. 2014;14(1):1-9.

64. Sarkar A, Yoo JY, Valeria Ozorio Dutra S, Morgan KH, Groer M. The association between early-life gut microbiota and long-term health and diseases. J Clin Med. 2021;10(3):459.

65. Van de Vliet M, Joossens M. The resemblance between bacterial gut colonization in pigs and humans. Microorganisms. 2022;10(9):1831.

66. Sheppard SK, Maiden MC. The evolution of *Campylobacter jejuni* and *Campylobacter coli*. Cold Spring Harb Perspect Biol. 2015;7(8):a018119.

67. Asrat D, Hathaway A, Ekwall E. Studies on enteric campylobacteriosis in Tikur Anbessa and Ethio-Swedish children's hospital, Addis Ababa, Ethiopia. Ethiop Med J. 1999;37(2):71-84.

68. ~~Debelo M, Mohammed N, Tiruneh A, Tolosa T. Isolation, identification and antibiotic resistance profile of thermophilic *Campylobacter* species from Bovine, Knives and personnel at Jimma Town Abattoir, Ethiopia. PLoS One. 2022;17(10):e0276625.~~

69. Chanyalew Y, Asrat D, Amavisit P, Loongyai W. Prevalence and antimicrobial susceptibility of thermophilic *Campylobacter* isolated from sheep at Debre Birhan, North-Shoa, Ethiopia. Agric Nat Resour. 2013;47(4):551-60.

70. Kassa T, Gebre-Selassie S, Asrat D. Antimicrobial susceptibility patterns of thermotolerant *Campylobacter* strains isolated from food animals in Ethiopia. Vet Microbiol. 2007;119(1):82-7.

71. Lazou T, Houf K, Soultos N, Dovas C, Iossifidou E. *Campylobacter* in small ruminants at slaughter: prevalence, pulsotypes and antibiotic resistance. Int J Food Microbiol. 2014;173:54-61.

72. Kerkhof P-J, Peruzy MF, Murru N, Houf K. Wild boars as reservoir for *Campylobacter* and Arcobacter. Vet Microbiol. 2022;270:109462.

73. Harrison LM, Balan KV, Hiett KL, Babu US. Current methodologies and future direction of *Campylobacter* isolation and detection from food matrices, clinical samples, and the agricultural environment. J Microbiol Methods. 2022:106562.

74. Deblais L, Ojeda A, Brhane M, Mummed B, Hassen KA, Ahmedo BU, et al. Prevalence and Load of the *Campylobacter* Genus in Infants and Associated Household Contacts in Rural Eastern Ethiopia: a Longitudinal Study from the *Campylobacter* Genomics and Environmental Enteric Dysfunction (CAGED) Project. Appl Environ Microbiol. 2023:e00424-23.

75. Giacomelli M, Follador N, Coppola L, Martini M, Piccirillo A. Survey of *Campylobacter* spp. in owned and unowned dogs and cats in Northern Italy. Vet J. 2015;204(3):333-7.

76. LaLonde-Paul D, Cummings KJ, Rodriguez-Rivera LD, Wu J, Lawhon SD. Ciprofloxacin resistance among *Campylobacter jejuni* isolates obtained from shelter dogs in Texas. Zoonoses Public Health. 2019;66(3):337-42.

77. Damborg P, Olsen KE, Møller Nielsen E, Guardabassi L. Occurrence of *Campylobacter* jejuni in pets living with human patients infected with C. jejuni. J Clin Microbiol. 2004;42(3):1363-4.

78. Heimesaat MM, Backert S, Alter T, Bereswill S. Human campylobacteriosis-A serious infectious threat in a One Health perspective. Fighting *Campylobacter* infections: Towards a One Health Approach. 2021:1-23.

79. Thépault A, Rose V, Queguiner M, Chemaly M, Rivoal K. Dogs and cats: reservoirs for highly diverse *Campylobacter jejuni* and a potential source of human exposure. Anim. 2020;10(5):838.

~~80. Vasco K, Graham JP, Trueba G. Detection of zoonotic enteropathogens in children and domestic animals in a semirural community in Ecuador. Appl Environ Microbiol. 2016;82(14):4218-24.~~

81. European Food Safety Authority (EFSA), European Centre for Disease Prevention and Control (ECDC). The European Union Summary Report on Antimicrobial Resistance in zoonotic and indicator bacteria from humans, animals, and food in 2020/2021. EFSA J. 2023;21(3):e07867.

82. Centers for Disease Control and Prevention (CDC).Antibiotic resistance threats in the United States, 2019. Web citation: https://www cdc gov/drugresistance/biggest-threats html Accessed. 2021;20.

83. Adiguzel MC, Sigirci BD, Celik B, Kahraman BB, Metiner K, Ikiz S, et al. Phenotypic and genotypic examination of antimicrobial resistance in thermophilic *Campylobacter* species isolated from poultry in Turkey. J Vet Res. 2018;62(4):463.

84. Food, Medicine, and Health Care Adminstration and Control Authority of Ethiopia. Standard Treatment Guideline for Health Center, 3rd ed. http://wwwfmhacagovet/wp-content/uploads/2019/03/STG-Health-Centerpdf.

85. Pavlova M, Alexandrova E, Donkov G, Mitova-Mineva Y, Kantardjiev T, Velev V. Campylobacter infections among Bulgarian children: molecular characterization and antimicrobial susceptibility. Biotechnol Biotechnol Equip. 2020;34(1):1038-42.

86. Wieczorek K, Wołkowicz T, Osek J. Antimicrobial resistance and virulence-associated traits of *Campylobacter jejuni* isolated from poultry food chain and humans with diarrhea. Front Microbiol. 2018;9:1508.

87. Bae W, Kaya KN, Hancock DD, Call DR, Park YH, Besser TE. Prevalence and antimicrobial resistance of thermophilic *Campylobacter* spp. from cattle farms in Washington State. Appl Environ Microbiol. 2005;71(1):169-74.

88. Valenza G, Frosch M, Abele-Horn M. Antimicrobial susceptibility of clinical *Campylobacter* isolates collected at a German University hospital during the period 2006–2008. Scand J Infect Dis. 2010;42(1):57-60.

89. Abd El-Ghany WA. One Health approach of campylobacteriosis in Egypt: An emerging zoonotic disease. J Infect Dev Ctries. 2019;13(11):956-60.

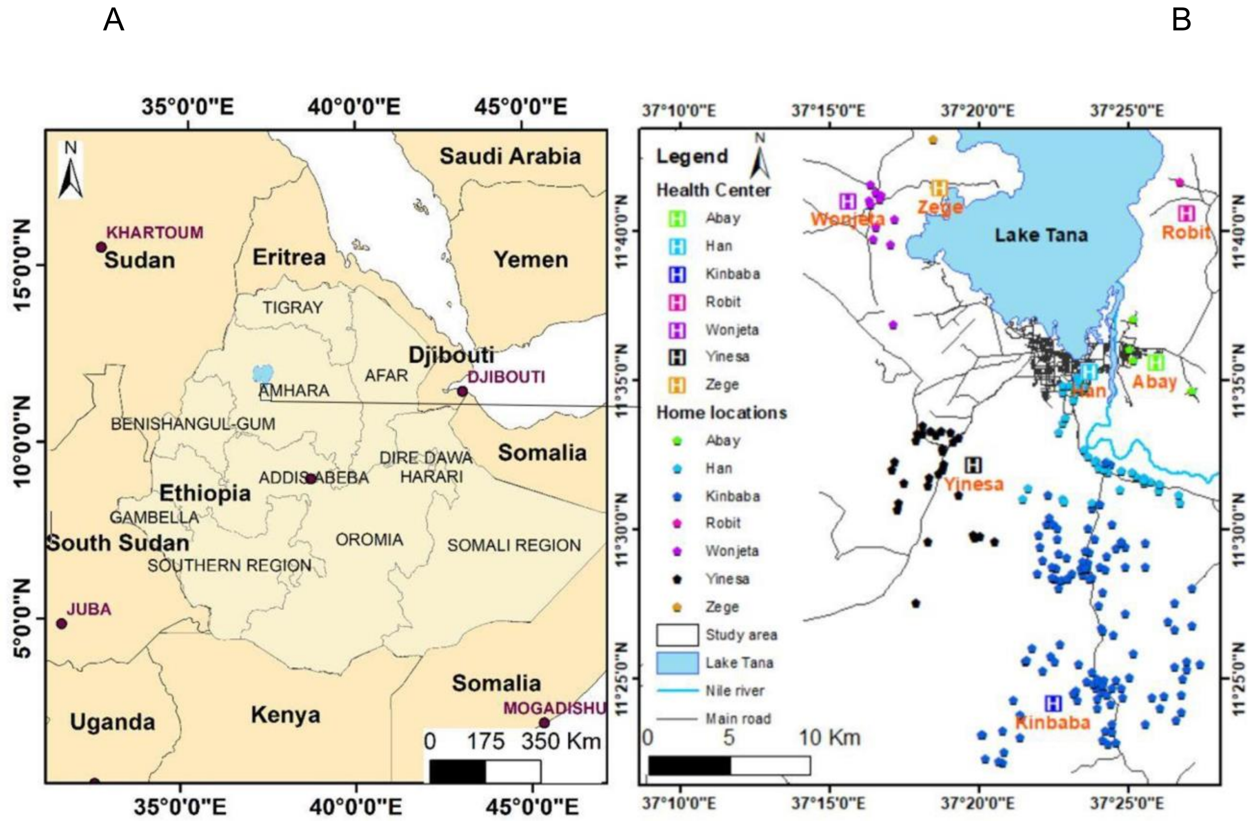


Fig 1. (A) Location map of the study area. (B) spatial distribution of health centers and sampled households

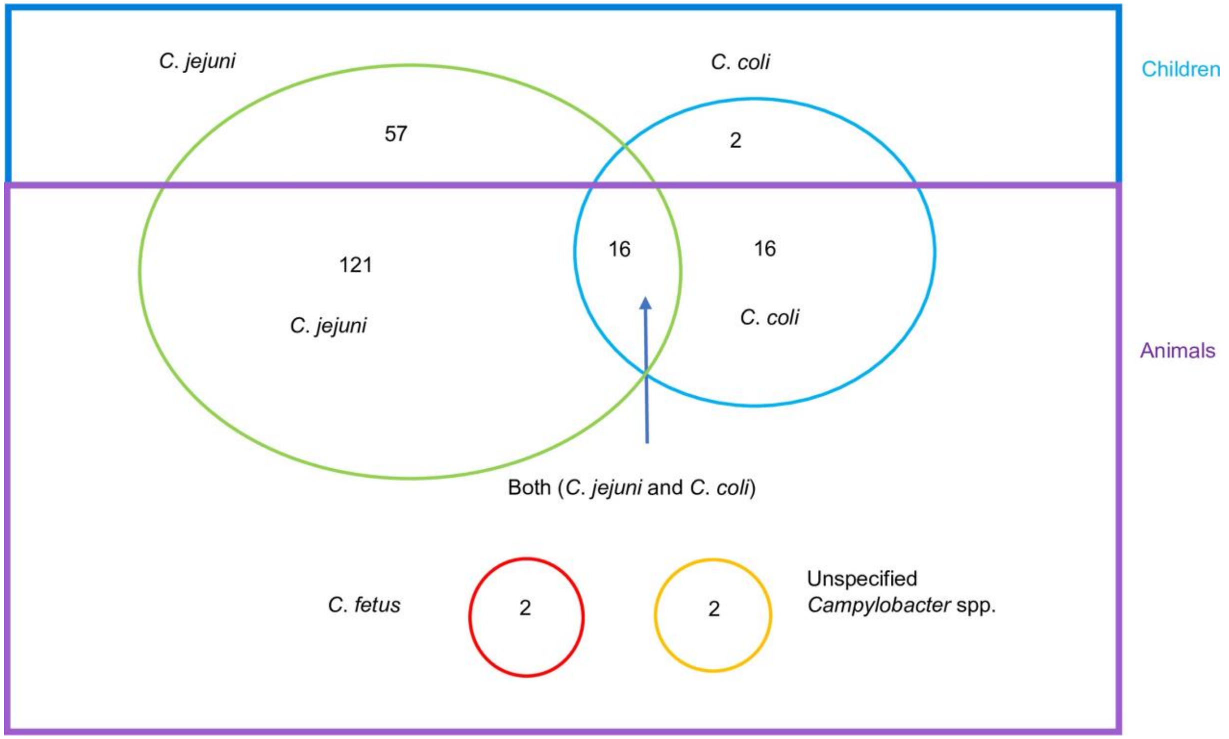


**Fig 2. Prevalence of *Campylobacter* species in children and animals.**

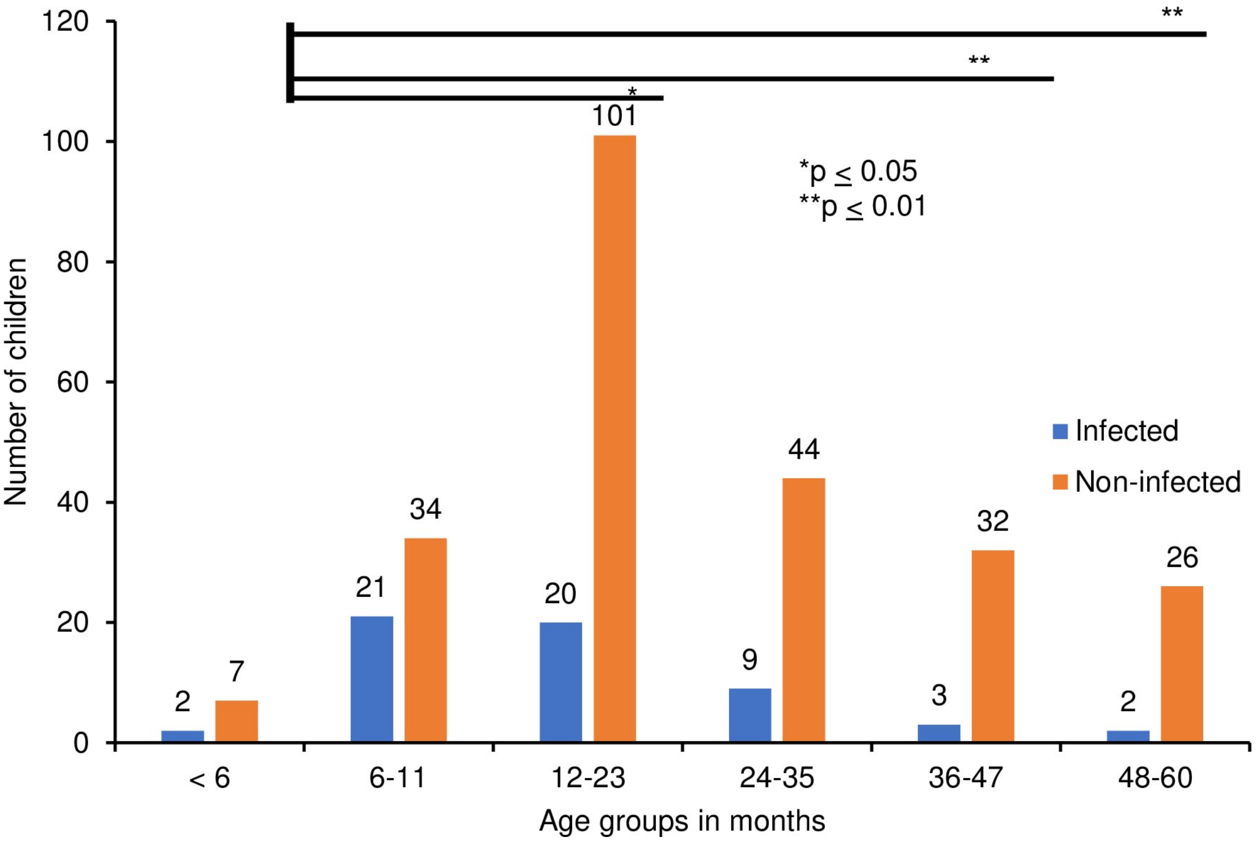


**Fig 3. *Campylobacter* infection per age group of children.**

Based on Games-Howell post hoc test multiple comparisons, the proportion of *Campylobacter* infection was significantly higher in age groups between 6 to 11 months compared to 12 to 23 months ($p = 0.05$), 36 to 47 months ($p = 0.006$) and 48 to 60 months ($p = 0.004$). No significant difference was observed between the age-group 6 to 11 months and the remaining age-groups ($p > 0.05$).

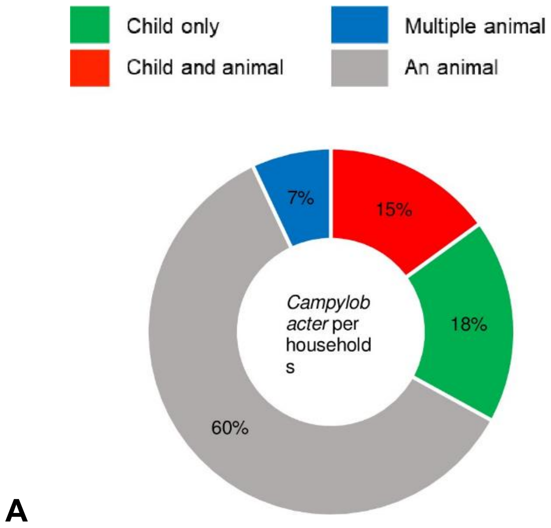


*Campylobacter* was present in at least one host in 61% of 245 households studied. The detection of *Campylobacter* in an animal species represents 60% of the occurrences per households, followed by 18% detection only in the child, 15% in both a child and animal species, and 7% in multiple animal

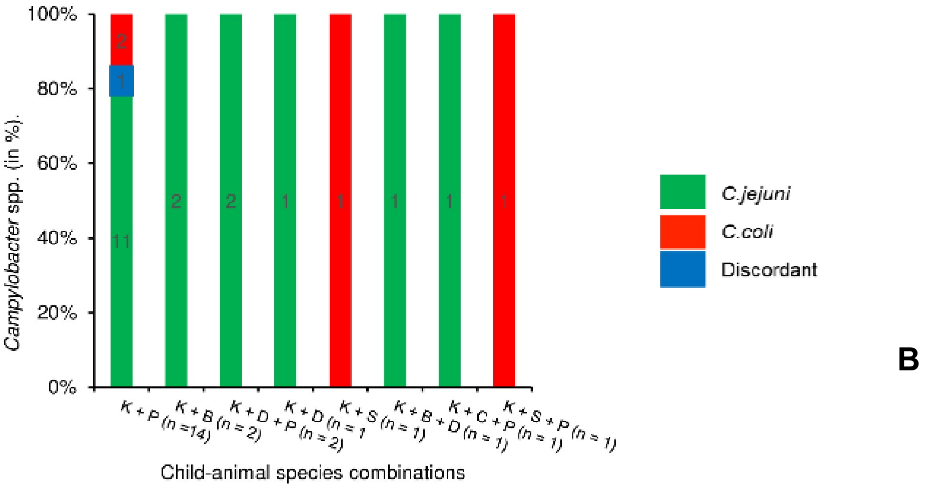


B, Bovine; C, Calf; D, Dog; K, Child; P, Poultry; S, Sheep

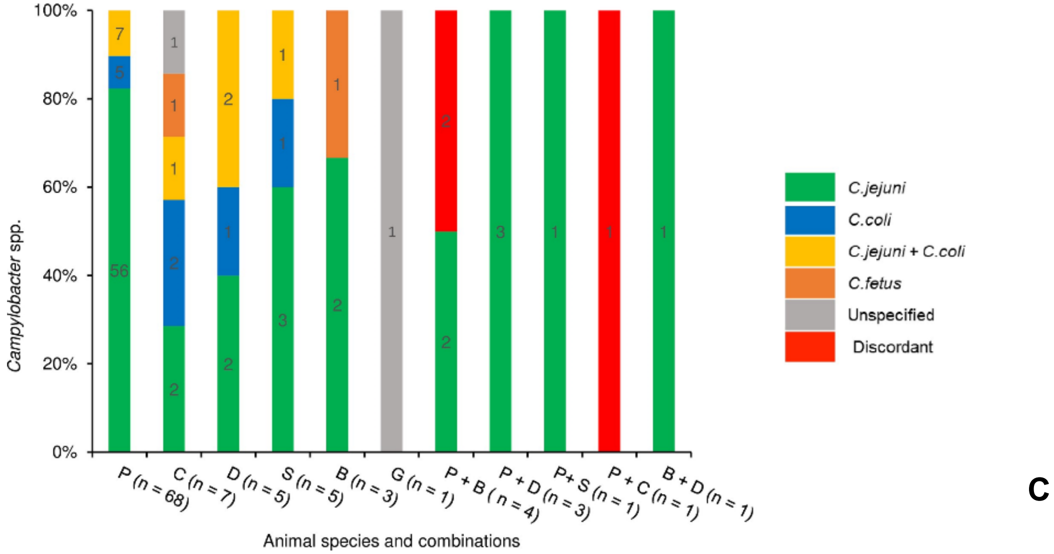


B, Bovine; C, Calf; D, Dog; K, Child; P, Poultry; S, Sheep;

**Fig 4. Occurrences of *Campylobacter* per 245 households in (A), *Campylobacter* species in child-animals combinations (n = 23) in (B), and per animal species and combinations in (C) (n = 99)**
