# Supplementary material for: Campylobacter occurrence and antimicrobial resistance profile in under five-year-old diarrheal children, backyard farm animals, and companion pets
Source: PLoS Negl Trop Dis. 2024 Jun 4;18(6):e0012241. doi: 10.1371/journal.pntd.0012241 (PMC11178231; doi:10.1371/journal.pntd.0012241)
Supplement: Attachment — Submitted filename: Rebuttal letter.docx [file pntd.0012241.s008.docx]

**Point-by-point responses to the comments of reviewer 1**

Dear reviewer, we sincerely appreciate your feedback and constructive comments. All the comments and suggestions are carefully addressed, and the updated areas are highlighted in yellow. Point-by-point responses to each comments and suggestion are detailed below.

*The goal of this study, to use new methods to quantify Campylobacter in young children experiencing diarrheal disease and in animals in Ethiopia is well articulated. The methods seem appropriate to address this objective and the populations which were sampled are well described. However, it is unclear if all animals in the home were sampled or if only a subset were sampled. If the data is available, information about children who were not eligible to participate due to insufficient sample collection would be useful to include as well.*

**Response**: If a household contains various animal species such as poultry, bovine, sheep, and goats, we collected a representative sample from each species. If a particular animal species has a small number, we collect samples from all individuals. Conversely, if the household has multiple animals of the same species, we sampled part of them, assuming shared contamination sources within the same compound. (now added in the text).

*The analysis matches the methods described.*

*The structure of Table 1, with the different sections for occurrence and identification could be more clearly presented, as in the current form the difference between the two sections is not immediately clear.*

***Response****:* Table 1 was indeed a bit confusing, and therefore in the revised manuscript, data in table 1 has been rearranged, presenting the total number of *Campylobacter* positive samples, as well as the species distribution.

*Overall the description of the results is comprehensive. However, in the section on demographic data in children, it is mentioned that there is no association between infection and number of siblings but no additional information is provided in Table 2 or in the text.*

**Response:** Relevant details regarding the lack of association between Campylobacter infection and the number of siblings have been incorporated into the revised manuscript text (highlighted in yellow).

*In Table 5 is also unclear why these specific sample pairs were selected to be presented. Based on Figure 4, they do not appear to be all instances of these concurrent isolations.*

***Response****:* we acknowledge that not all instances displayed in Figure 4 are also presented in Table 5. This is because antimicrobial susceptibility testing (AST) was not conducted for instances no isolate was available (only positive by PCR detection). Therefore, the sample pairs presented in Table 5 represent only a subset of those in Figure 4. This selection enables a comparison of the AST profiles of *C. jejuni* isolates between children and animals from the same home.

*The authors conclude that these results suggest direct animal to human transmission. However, given the cross-sectional nature of this study, concluding transmission instead of simply co-occurrence in both groups is a not supported by the data. In addition, the unmatched AST profiles between animals and children support the possibility of other explanations, which the authors do mention later in the discussion. Discussion of limitations is minimal and could be further expanded upon to include limitations other than those related to molecular methods. The authors discuss their findings in the context of previous work done both in Ethiopia and globally. However, further consideration of reasons for differences in findings beyond culture techniques could be discussed if they exist. In the one study where this is discussed, the differences in methods with regards to who was sampled are significant, which raises the question of the inclusion criteria in other studies which are discussed. Finally, while Campylobacter infection is arguably a One Health issue, and this study makes reference to One Health at several points,* *this study does not include all three domains of One Health.* *It does not include environmental samples and includes minimal discussion of the role of the environment in transmission or as an opportunity for intervention.*

**Response**: Indeed, the cross-sectional nature of the study does not allow concluding direct transmission from animals to children. Therefore, we have modified our conclusion to reflect the possibility of transmission at the household-level, rather than direct transmission from animals to children (changed in the text).

We have discussed the limitation of the cross-sectional nature of this study and the absence of genetic analysis in identifying transmission dynamics between animal species and children in the revised manuscript. The need for further longitudinal studies to ascertain causality is also recommended in the revised manuscript.

We appreciate your observation regarding the unmatched AST profiles between children and animals. We have revised the manuscript to indicate that the matched AST profiles between children and animals in some homes may support household-level transmission, while unmatched profiles in other homes may indicate the complexity of the *Campylobacter* ecology and transmission dynamics. This is discussed in the revised manuscript.

Regarding the reasons for differences between our findings and findings in the context of previous work done both in Ethiopia and globally, we have thoroughly examined various reasons beyond the difference in the culture techniques. Variations in methods of children recruitment (health center-based versus house-to-house search), definition of diarrhea, type of diarrhea (acute or persistent), and admission (admitted versus outpatient) have been discussed in the revised manuscript. Furthermore, we have discussed variations in the status of malnutrition, immunodeficiency, child-dwelling settings (urban versus rural), and sanitation and hygiene levels.

Regarding the gaps on the three domains of One Health, due to logistical constraints and feasibility issue, environmental samples such as surfaces or dust, and water sources where children and animals interact together were not investigated. As a result, the environmental domain of One Health is less addressed in this study and the role of environment in transmission or as an opportunity for intervention discussed minimally. These limitations are now mentioned in the revised manuscript. We have reduced sections referencing One Health and clarified that this study addressed only the human and veterinary domains. Furthermore, discussion on limitations beyond molecular methods are expanded in the revised manuscript.

*In the results section on child demographics, reference is made to as group classifications of the Ethiopian Public Health Institute. However, the phrasing of this significant difference is unclear and could be reworded or clarified. The first sentence in the discussion also states that the study presents the spatial distribution of Campylobacter. However, while the spatial distribution of sampled households is presented, those that were positive are not delineated.*

**Response**: We have now rephrased and clarified the differences in *Campylobacter* occurrence between the different age groups. Additionally, we have incorporated a figure (Fig 5) showing the spatial distribution of households with and without *Campylobacter* in the revised manuscript. Furthermore, a brief description of the spatial distribution has been incorporated into the results section of the revised manuscript.

*In general, this study presents useful information about the burden of Campylobacter in the study region. It's strengths include it's methods and the number of samples, as well as the description of the distribution and characteristic of positive samples. The authors adequately discuss these findings in the context of previous findings. However, the conclusions drawn from the study regarding transmission are ambitious given the cross sectional nature of the study and lack of genetic analysis. The limitations of this study and how it fits into a One Health approach could also be expanded. In addition, there are places where the writing could be reworded to improve clarity.*

**Response:** Considering the limitations of cross-sectional nature of the study and the absence of genetic analysis, we have now modified the conclusions to better reflect the findings of this study. We have expanded the potential limitations of this study such as the cross-sectional nature of the study, lack of genetic analysis, and others in the revised manuscript. Moreover, we have revised the manuscript to show that our study addresses only the human and veterinary domain of One Health. Additionally, some texts and graphs have been reviewed and reworded and the clarity of the manuscript is much improved. All the changes are highlighted in yellow.

**Reviewer 2**

Dear reviewer, we sincerely appreciate your feedback and constructive comments on the manuscript. All comments and suggestions are carefully addressed in the revised manuscript. The updated areas of the manuscript are highlighted in yellow. Point-by-point responses to both general and specific comments provided below, as well as integrated within the main document are detailed below.

*The paper may require language edition. The manuscript would benefit from re-visiting it to a recently accepted manuscript template. While the broad/main objective has been clearly articulated in the introduction part, there are no specific objectives, research questions, and a hypothesis partly in the ‘Abstract’ and introduction. These are needed to guide the analyses you made as well as to anchor the results and discussion.*

**Response:** we thoroughly revised the manuscript for the language, and revisited the manuscript using a recently accepted manuscript template. We have incorporated specific objectives and hypotheses into the abstract and introduction sections of the revised manuscript.

***Specific comments***

***Title****: Campylobacter in children under the age of five with diarrhea, backyard farm animals, and companion animals: Occurrence and antibiotic resistance*

*Suggest: Occurrence and antibiotic resistance profile of Campylobacter among under five-year diarrheic children, farm- and companion- animals or Campylobacter occurrence and antibiotic resistance profile in under five-year-old diarrheal children, farm animals, and companion animal*

**Response**: After careful consideration, we have accepted the second suggestion. However, we have chosen to retain the term “backyard” to specify the type of farm animals sampled in this study. The animals included in this study were raised in backyards for personal consumption and daily farm activities rather than for commercial purposes. While the term “Farm animals” usually encompasses those raised on a large scale for commercial purposes, we believe that “backyard farm animals“ better reflects our study subjects than the broad “farm animal” alone. Therefore, we make the title “*Campylobacter* occurrence and antimicrobial resistance profile in under-five-year-old diarrheal children, backyard farm animals and companion pets”.

***Abstract***

*Suggest: developing country for “growing countries”*

**Response**: we changed into “low-income countries” instead of “developing countries” as it has less negative connotation, and is more internationally applied nowadays.

*Suggest: Antimicrobial resistance (AMR) is preferable as it is used on page 4, second paragraph than Antibiotic resistance (ABR). Revise throughout to adjust uniformly to AMR since both are used in your manuscript*

**Response**: The term “antibiotic resistance” is replaced with “antimicrobial resistance (AMR)” in the revised manuscript. These changes are controlled throughout the manuscript.

***Introduction:*** *1st paragraph first line the dot (‘.’) be changed to the acceptable comma (‘,’) form “….. 525,000 deaths ….”. There are similar patterns throughout and check if they have to be changed. Page 6: first paragraph second line: citations for the standard guidelines are not indicated.*

**Response**: We have replaced (‘.’) with commas (‘,’) throughout the manuscript. Additionally, we have included a citation for the standard guideline in the first paragraph of page 6 in the revised manuscript.

***Study area and design***

*According to the details provided under the subtitle "Study participants," the study is conducted at a healthcare facility, and the participants were people seeking medical attention at one of the many Bahir Dar healthcare facilities. Because most symptomatic instances of campylobacter may not seek medical attention, the study cannot be considered community-based.*

**Response**: the study design is changed from “community-based” to “healthcare facility-based” in the revised manuscript.

***Isolation and preliminary identification of Campylobacter***

*Page 7: The isolation of campylobacter was carried out in this subtitle for a maximum of five days at 37 degrees Celsius in a microaerobic atmosphere (burning candle). Under the same conditions, additional confirmation was carried out in an Ethiopian laboratory using the candle extinction method. Although the researchers used sophisticated identification techniques in Belgium and appropriate re-cultivation techniques for campylobacter, the initial isolation methods used in Ethiopia—that is, the candle extinction method for primary isolation versus the maintenance of microaerobic conditions through the introduction of a gas generator to provide a mixture of gases (10% CO2, 10% H2, and 80% N2) under the heading “Re-cultivation of presumptive Campylobacter isolates”—cannot provide confidence for the true rate of campylobacter positivity.*

**Response**: We acknowledge that the candle extinction method, may not provide the same level of confidence in isolating *Campylobacter* compared to more sophisticated techniques, though it is easily applicable in the field. Our study favorably isolated *C*. *jejuni* and *C*. *coli*, major causes of gastrointestinal *Campylobacter* infection. We have included the limitations of the candle extinction method, particularly in isolating low numbers or slow-growing *Campylobacter* cells, in the revised manuscript.

*The initial measures taken to recover Campylobacter from the specimens of symptomatic children and the different animals revealed one of the study's major weaknesses. It is evident that the bacteria are very sensitive to even a small change in environmental factors. According to the majority of the literatures, microaerophilic bacteria, such as Campylobacters, can be grown in plastic bags or sealed jars using commercial gas-generating envelopes that contain mixes of CO2, H2, O2, and N2.*

**Response**: the remark on the sensitivity of *Campylobacter* towards the cultering condition is correct, though applies more when the bacteria is isolated from food or environmental matrices in which *Campylobacter* is in stressed condition. In clinical microbiology (human and veterinary), enrichment is not applied (also not in Europe), and the incorporation of H_2_ in the gas mixture is not a routine setting. Even more, the candle method has shown to reach a much faster microaerobic condition, then when gas packs are applied (usually the latter takes 4 h or more, in contrast to a few minutes with the candle).

*Table 2: The ‘Age’ column addition is 57, and further the total children involved become 301. Where are the two positive results missed in the table2?*

**Response**: during the data collection period, the exact ages of 2 out of 303 children were not recorded. Consequently, these two children were excluded in the age category, resulting in a total of 301 children for this parameter. Hence, the addition for the age column is 57 instead of 59, and the total becomes 301 for this particular variable. This clarification is incorporated in the description section of Table 3.

*Table 4: Break point; 0.004*

**Response**: Thank you. Corrected in Table 5 in the revised manuscript.

References (page 29-38): The number of references is too much. There is redundant citation of the same article in four places as can be checked from the PDF file.

**Response**: We have corrected all instances of redundant citations in the revised manuscript, and reduced the number of references.

**Questions for consideration:**

1. *Incubation of cultured media for Campylobacter isolation use to be for 48 hours with additional 24 hours before it is recorded negative. However, in your study, it was extended for up to five days. How much of your isolates were recovered by extending to 72 to 120 hours before it was defined as ‘No growth for Campy”? That part of knowledge is essential.*

**Response**: the majority of *Campylobacter* isolates were obtained within the first 48-hour of incubation period, but the exact number of additional positives after more then 48 h was not exactly recorded, but for sure, there were only few of them.

*2. In discussion part, page 23, second paragraph last line: “…. the use of microaerobic conditions without hydrogen during isolation” was stated as a reason for failing to recover slow-growing campylobacter species. Don’t you believe that you also missed the human important fast growing campylobacter pathogens from your study?*

**Response**: we do not believe that the human important fast growing *Campylobacter* pathogens are missed by the absence of hydrogen, as it has been shown in literature that *C*. *jejuni* and *C*. *coli* do not need hydrogen. That is why we highlighted the impact of the absence of hydrogen in the candle jar microaerobic incubations on isolating slow-growing *Campylobacter* species.

*Which Campylobacter species that may be relevant in your study may be missed by the absence of the gas as well?*

**Response**: It could be, but not well documented in literature that less common slow-growing emerging *Campylobacter* species such as *C. lari,*  *C*. *upsaliensis*, *C*. *hominis*, *C. hyointstinalis*, *C. concisus*, *C. rectus*, *C. curvus*, *C. helveticus,* and *C. urealyticus* might have been missed by the absence of gas.

*3. Which statistical tools are used for which variables, parameters or factors cannot be appreciated in the manuscript. As some of the observed values are too small to extract meaningful interpretation and conclusion, a reliable professional consultation may be necessary.*

**Response:** We have now more specified the statistical tools used for each variable examined in the manuscript. A chi-Square test was used to evaluate the presence of significant associations between categorical variables (such as gender, different age groups, frequency of stool per day, and duration of diarrhea) and *Campylobacter* infection in children (classified as infected or non-infected). Similarly, the presence of a significant associations between each of the symptoms related to diarrhea (including watery diarrhea, foul-smelling diarrhea, vomiting, abdominal pain, and fever) and *Campylobacter* infection was examined using the chi-Square test. Additionally, the Fisher’s exact test was used to evaluate the difference in *Campylobacter* infection between children with or without bloody stool, due to one of the observed values falling below 5.

The normality of the continuous data such as age of the children in months, frequency of diarrhea per day, duration of diarrhea, and number of siblings per household, was tested by both the Shapiro-Wilk and Kolmogorov-Smirnov tests. All these continuous data are asymmetrical. As a result, medians with interquartile ranges were calculated to compare the groups, and differences were assessed by non-parametric statistical tests, Mann-Whitney U test. To compare the median ages of children with and without *Campylobacter* infection, the Mann-Whitney U test was calculated. Likewise, Mann-Whitney U test was applied to compare the median duration of diarrhea, the median frequency of diarrhea, as well as the median number of siblings, between *Campylobacter*-infected and non-infected children. The above details are well articulated in the revised manuscript.

**Response to specific comments written by the reviewer from the document**

***Abstract***

*“new” recent or improved? What is new?*

**Response**: The word “new” is replaced with the word “improved” in the revised manuscript.

**Responses in the Introduction section**

- We have incorporated the term “years” next to the text “children under the age of five” in the concluding part of the introduction.
- A reference citation is added for the standard guideline in the concluding part of the introduction .

***Materials and Methods***

*Children experiencing diarrhea for more than seven days and coming from a household without any backyard farm animals were excluded. How about pet animals while farm animals are absent?*

**Response**: we specifically targeted diarrheal children from households that own at least one backyard farm animal. This decision was made because some homes may not have companion pets, or if they do, they typically have limited numbers. Additionally, obtaining fecal samples from companion pets has practical challenges. For example, cats often bury their feces in soil, making collection difficult, while dogs have the freedom to roam in their environment, complicating the sampling process. Therefore, in order to maintain consistency and address the interface between children and animals, we excluded households without at least one backyard farm animal, regardless of the presence of companion pets.

*Based on the study participants information, it is healthcare facility based research where the study participants were health seekers coming to the health centers, yes?*

**Response**: all children included in our study were examined for diarrheal illness at health centers, where we collected stool samples from them. Hence, the accurate description of our study design is healthcare facility-based research. This modification has been incorporated into the revised manuscript. In addition, the symbol for easting (“E”) is added to the study area description.

*Relevance of mentioning “Health Centers that were logistically difficult to access and had only few children with diarrhea during the collection period of the current study were excluded” does not show benefit.*

**Response:**  we acknowledge that the exclusion criteria related to health care centers with limited accessibility and a low incidence of diarrheal cases might not immediately showcase their benefits in the study narrative. Therefore, it is omitted in the revised manuscript.

*You may replace the blue highlights "From each of the health centers within a 0.4 to 12.8 KM radius, every household with the suspected cases was visited."*

**Response**: The sentence “The houses visited in this Bahir Dar City recruitment area were in a radius of 0.4 to 12.8 kilometers from the health centers. In Kinbaba, the houses visited were in a 1.5 to 12.8 km radius from the health center; in Yinesa, they were in a 1.7 to 9.2 km radius; and in Wonjeta, they were in a 1.3 to 8.3 km radius.” is replaced with the text "From each of the health centers within a 0.4 to 12.8 km radius, every household with the suspected cases was visited" in the revised manuscript.

*In the stool sample and data collection from children, Where is the tool? Which important variables are otherwise addressed in the stool?*

**Response**: The text ”according to the approved tool” inserted by mistake is deleted in the revised manuscript. This is because data for the variables age, gender, number of siblings, and symptoms related to diarrhea were collected at the health center while the children were examined for their diarrheal illness. Therefore, the text according to the approved questionnaire is omitted in the revised manuscript.

*Was there a kind of pooling fecal samples for the respective animal species type per household? Or did you use a different sampling approach to select the animals in a household?*

**Response**:. Our sampling strategy involved a combination of approaches based on the specific circumstances of each animal species and household dynamics. Pooled sampling, or sampling from subsets, was utilized for both poultry and bovine. This sampling approach was similarly employed for sheep and goats. Samples from adults and calves were collected separately in the case of bovine. Households typically had only one or a few claves. Due to their typical presence in singular quantities per home, fecal samples were collected from one companion animal for the majority of homes sampled. This adaptability allowed us to address diverse household scenarios, accommodating both comprehensive sampling and sampling of subsets. This sampling strategy is mentioned in the revised manuscript.

**Response**: In the isolation and preliminary identification of *Campylobacter* section:

- Standard microbiological practices instead of “procedures”
- The word “about” is added before the term “1 gram”
- The term “homogenized” instead of “diluted” is used
- The abbreviation “Ltd” is included in the revised manuscript

*An additional aliquot was kept at -20 ^°^C in Eppendorf tubes with 1 ml of sterile physiological water. Is this without cryoprotective solution/solvent at lower temperature? Please cite a relevant method of following these methods.*

**Response**: we described in the method section: pure colonies with typical *Campylobacter* morphology were stored in cryotubes with 1 ml of sterile sheep blood at -80 ^O^C.

Additional aliquots were swabbed in 0.85% physiological sterile water and stored at -20 ^O^C. We did not use cryoprotective solutions or solvents such as glycerol to protect *Campylobacter* cells from damage during freezing, as the intention was to use this as a backup for DNA detection if the *Campylobacter* cells stored in 1% sheep blood were stressed and did not recultivate.

*PCR-based analysis of genus and species of Campylobacter -------------- alternative subtitle*

**Response**: title has changed according to your suggestion.

***Results***

*In Table 2, how the p value for the median age is calculated*

**Response**. The age of the children in months is a non-normally distributed continuous data among *Campylobacter* infected and non-infected children. As a result, the median value with IQR was calculated to compare the infected and non-infected children. To assess, the significant differences in the median age of the infected and non-infected children, the Mann-Whitney U test was analyzed, and the p-value was < 0.001. Questions related to the statistical tests employed are addressed in the method section of the revised manuscript.

**Responses to specific points in the discussion**

**First paragraph of the discussion**

- The term highlighted in yellow in the first paragraph of the discussion “conventional microaerobic incubation system” is replaced with the term ” burning candle microaerobic incubation system”.
- The term "compared to earlier regional studies", is changed into "compared to earlier studies in Ethiopia"

**Second paragraph of the discussion**

- Changed "no specialist laboratories" to "restricted resources for laboratories".
- Replaced "poultry" and "dogs" with “backyard farm animals" and "companion pets".

**On page 22, paragraph 2,**

- Reworded “ between the ages of 6 and 11 months to “ from 6 to 11 months.
- Specified "the two oldest age groups" as "from 36 to 47 months of age" and "from 48 to 60 months of age"
- Added a p-value
- Incorporated the suggested text” earlier exposure to *Campylobacter* induces specific partially protective immunity”

**Page 24, second paragraph:**

- Replaced “eating” with “dining”.
- Changed “unrestrained” to “unrestricted”.

**Page 25, third paragraph**

- Replaced “cross-resistance with “co-resistance”
